# Proinflammatory oscillations over the menstrual cycle drives bystander CD4 *T* cell recruitment and SHIV susceptibility from vaginal challenge

**DOI:** 10.1016/j.ebiom.2021.103472

**Published:** 2021-07-03

**Authors:** Alison Swaims-Kohlmeier, Anandi N. Sheth, Jed Brody, Felicia P. Hardnett, Sunita Sharma, Erin Wells Bonning, Igho Ofotokun, Ivana Massud, J.Gerardo García-Lerma

**Affiliations:** a Division of HIV/AIDS Prevention, National Center for HIV/AIDS, Viral Hepatitis, STD, and TB Prevention, Centers for Disease Control and Prevention, Atlanta, GA 30329, United States.; b Division of Infectious Diseases, Department of Medicine, Emory University, Atlanta, GA 30322, United States; c Department of Physics, Emory University, Atlanta, GA 30322, United States

**Keywords:** HIV risk-factors, Menstrual cycle, Macaque models, Female reproductive tract

## Abstract

**Background:**

The menstrual cycle influences HIV infection-risk in women, although the timing and underlying mechanism are unclear. Here we investigated the contribution of the menstrual cycle to HIV susceptibility through evaluating immune behavior with infection-risk over time.

**Methods:**

Blood and vaginal lavage samples were collected from 18 pig-tailed macaques to evaluate immune changes over reproductive cycles, and from 5 additional animals undergoing repeated vaginal exposures to simian HIV (SHIV). Peripheral blood mononuclear cell (PBMC) samples from healthy women (*n* = 10) were prospectively collected over the course of a menstrual cycle to profile *T* cell populations. Immune properties from PBMC and vaginal lavage samples were measured by flow cytometry. Plasma progesterone was measured by enzyme immunoassay. The oscillation frequency of progesterone concentration and CCR5 expression on CD4 *T* cells was calculated using the Lomb-Scargle periodogram. SHIV infection was monitored in plasma by RT-PCR. Immune measures were compared using generalized estimating equations (GEE).

**Findings:**

Macaques cycle-phases were associated with fluctuations in systemic immune properties and a type-1 inflammatory *T* cell response with corresponding CCR5+ memory CD4 *T* cell (HIV target cell) infiltration into the vaginal lumen at the late luteal phase. Power spectral analysis identified CCR5 oscillation frequencies synchronized with reproductive cycles. In a repetitive low-dose vaginal challenge model, productive SHIV_163P3_ infection only occurred during intervals of mounting type-1 *T* cell responses (*n* = 5/5). Finally, we identify similar type-1 inflammatory *T* cell responses over the menstrual cycle are occurring in healthy women.

**Interpretation:**

These data demonstrate that periodic shifts in the immune landscape under menstrual cycle regulation drives bystander CCR5+ CD4 *T* cell recruitment and HIV susceptibility in the female reproductive tract.

**Funding:**

This study was supported by the U.S. Centers for Disease Control and Prevention, Atlanta, GA 30329 and NIH grants to Emory University (K23AI114407 to A.N.S., the Emory University Center for AIDS research [P30AI050409], and Atlanta Clinical and Translational Sciences Institute [KLR2TR000455, UL1TR000454]).

**Disclaimer:**

The findings and conclusions in this report are those of the authors and do not necessarily represent the views of the U.S. Centers for Disease Control and Prevention or the Department of Health and Human Services.

**Research in context**

**Evidence before this study**

Understanding the contribution of the menstrual cycle to STI/HIV infection from sexual contact may help prevent new infections, and inform upon other potential risk factors in women such as hormonal contraception. Exposure to HIV in the female reproductive tract (FRT) during fertility windows has been previously proposed to increase infection susceptibility through heightened immune tolerance. Although proinflammatory responses and the proximity of CCR5+ CD4 *T* cells (HIV target cells) to mucosal barriers are considered HIV-risk factors, when and how the immune system is regulated by the menstrual cycle to influence infection in the FRT remains undefined.

**Added value of this study**

We identify in humans and pig-tailed macaques that the menstrual cycle regulates a type-1 specific adaptive immune response, identified in part by increased CD4 *T* cell expression of proinflammatory properties and the HIV co-receptor CCR5 within the late luteal phase. Furthermore, in macaques we find that the sinusoidal fluctuations of CCR5+ memory CD4 *T* cells in circulation corresponds with infiltration of CCR5+ CD4 *T* cells into the vaginal mucosa primarily within the late luteal phase of the cycle, when SHIV inoculation also led to the establishment of infection from weekly repetitive low-dose vaginal challenge.

**Implications of all the available evidence**

This study finds evidence that over the menstrual cycle HIV-risk from sexual contact is increased by the FRT tissue remodeling functions that occur during the late luteal phase of the menstrual cycle.

Alt-text: Unlabelled box

## 1 Introduction

Despite the discovery and implementation of antiretroviral drugs to prevent and treat Human Immunodeficiency virus (HIV) infection, acquired immunodeficiency syndrome (AIDS)-related illness are still the leading cause of death globally in women aged 15–49 [1,2]. In 2017 it was estimated that the majority (79%) of new HIV infections in eastern and southern African adolescents occurred in women (340,000 new infections), who are disproportionally affected by gender-based abuses including young marriage and physical violence [1]. Globally, women comprised almost half of the 1.8 million new cases of HIV infection over that same year with the vast majority of transmissions likely occurring through sexual intercourse [2]. Based upon these statistics, HIV prevention strategies aimed at understanding and reducing HIV infection in women with a focus on heterosexual transmission events are a critical factor to combating the epidemic.

Although we have yet to understand the precise mechanisms by which HIV infection is established following mucosal exposure, the immune system is a principal component of the process. The localization of the main target of HIV infection, CCR5 expressing CD4 *T* cells, at mucosal tissue barrier sites of exposure is an indicator of susceptibility to HIV or simian immunodeficiency virus (SIV) in both human and non-human primates (NHP) [3], [4], [5], [6]. Moreover, the functional properties expressed by circulating memory CD4 *T* cells can also indicate the likelihood of establishing an infection. In some studies, HIV acquisition was found to correlate with the frequency of activation-associated properties and CCR5 from CD4 *T* cells in blood [[7], [8], [9], [10], [11]]. Thus, conditions that increase CCR5 and immune activation from both circulating and tissue resident CD4 *T* cells identify increased HIV infection risk.

It has long been recognized that preexisting inflammation, such as occurs in response to certain sexually transmitted infections (STIs) or bacterial vaginosis, increases the odds of HIV or SIV acquisition vaginally [[12], [13], [14]]. Such inflammatory immune responses are initiated through innate recognition of pathogenic molecular patterns which prompts proinflammatory cytokine/chemokine production at the induction site in order to recruit immune effector cells from the circulation [15,16]. Once engaged this defense must be further regulated in order to produce a coordinated immune response that effectively clears pathogen while preventing extensive tissue damage [17,18]. This regulation tailors the effector response into specific types which are broadly characterized according to the nature of the pathogen a response is mounted against [19].

Although the process of menstruation is not a result of any infection it comprises elements that are consistent with an inflammatory response. In menstruating mammals such as humans and some species of NHP, the unique function of spontaneous decidualization by endometrial stromal cells which are hypothesized to limit harmful levels of invasion by a potential embryo during implantation also demand extensive tissue remodeling of the uterine lining in order to maintain reproductive cycles [20]. This remodeling involves breakdown and shedding of the decidual cell layer and is mediated by infiltrating leukocytes from the circulation that are recruited through the local production of proinflammatory signals during the late luteal or late secretory phase of the menstrual cycle when ovarian progesterone production is decreased. While the process of menstruation is contingent upon the immune system, elucidating the mechanisms which regulate this response are challenged by a lack of model systems which experience menstruation comparable with humans [21,22].

Although an overwhelming majority of women of reproductive age globally including sub-Saharan Africa experience periodic menstruation [23] and though the menstrual cycle is implicated as a risk-factor for HIV infection from sexual exposure in women, the time frame of when increased risk occurs over the cycle and the underlying mechanisms governing this phenomena are unclear. A growing number of studies propose that under menstrual cycle regulation immune suppression during increased ovarian progesterone production increases HIV susceptibility in the female reproductive tract (FRT) [[24], [25], [26], [27], [28], [29]]. In this scenario, heightened immune tolerance necessary to facilitate potential blastocyst implantation reduces immune surveillance functions thus increasing infection opportunities. Moreover this time span of the cycle has been previously linked with increased susceptibility to *Chlamydia trachomatis, Candida albicans* and *Neisseria Gonorrhoeae* infections [[30], [31], [32], [33]]*.* However progesterone has an inhibitory effect on HIV infectivity [34,35] and *ex-vivo* FRT cross-sectional infection studies provide conflicting evidence as to whether HIV replication is more likely to occur at either the follicular or luteal phase [[36], [37], [38]]. In macaque studies productive infection with Simian-Human Immunodeficiency virus (SHIV) was more likely to occur from vaginal challenge during the late luteal phase [25,26], yet the mechanisms underlying this observation and whether key HIV-risk factors contributed to indicate applicability with humans was not investigated to our knowledge. Taken together these findings underscore critical gaps in our understanding of how the menstrual cycle can impact HIV infection and those key mechanisms that drive HIV-risk from sexual exposure.

To evaluate HIV susceptibility throughout the menstrual cycle we used the pig-tailed macaque (*Macaca nemestrina*) model as well as samples from healthy women to study immune properties with HIV-risk factors over time. Using this approach we identified systemic type-1 inflammatory shifts with CCR5 CD4 *T* cell infiltration into the vaginal mucosa most often occurring at the late luteal phase when progesterone production is decreased. Furthermore, we mapped cycle-associated variations in HIV risk factors by constructing periodograms of CD4 *T* cell expression of CCR5 over consecutive reproductive cycles to find novel sinusoidal components with frequencies coinciding with progesterone oscillations. We also document that productive SHIV infection in pig-tailed macaques correlates most strongly with rising CCR5 expression frequency, activation-associated properties, and proinflammatory responses by CD4 *T* cells. By monitoring healthy typical-cycling women over the course of a menstrual cycle we found that in addition to a type-1 inflammatory bias during the late luteal phase, CD4 *T* cell populations were more likely to express chemokine receptors that play a vital role in *T* cell trafficking into type-1 inflammatory signals. Our results demonstrate that menstrual cycle regulation of the immune system control inflammatory-driven trafficking properties by HIV target cells and can dictate infection opportunities in the female reproductive tract (FRT).

## 2 Methods

### 2.1 Animal ethics statement

A total of 18 pig-tailed macaques were used in this study. All procedures were approved by the Institutional CDC Animal Care and Use Committee (IACUC). Macaques were housed at the CDC under the full care of veterinarians in accordance with the Guide for the Care and Use of Laboratory Animals (National Research Council of the National Academies, 2010). All efforts were made to minimize suffering, improve housing conditions, and provide enrichment opportunities. Procedures were performed under anesthesia using standard doses of ketamine hydrochloride.

### 2.2 SHIV vaginal infection in pig-tailed macaques

The repeated low dose exposures model was used to measure susceptibility from vaginal challenge [39]. 50 tissue culture infectious doses (TCID_50_) of an R5-tropic SHIV_162P3_ isolate (obtained from the NIH AIDS Reagent Program) was added into the vaginal vault once a week for up to 15 weeks by non-traumatic inoculation. Challenges stopped once animal was determined SHIV RNA positive [40]. SHIV plasma RNA was quantified by RT-PCR as previously described [39]. The time of infection was defined as 7 days prior to RNA detection in plasma to account for the eclipse period between virus inoculation and detection of in infected macaques [41]. Virus-specific serologic responses were measured using a synthetic peptide enzyme immunoassay assay (BioRad, Genetic Systems HIV-1/HIV-2, Redmond, WA) [42].

### 2.3 Menstrual cycle monitoring

Weekly plasma samples were analyzed for progesterone concentrations at the University of Wisconsin National Primate Center using an enzyme immunoassay (Cayman Chemical; 57–83) [43].

### 2.4 Human ethics

This study was approved by the Emory University Institutional Review Board, the U.S. Centers for Disease Control and Prevention Institutional Review Board, and the Grady Research Oversight Committee. All participants provided informed consent.

### 2.5 Study participants

T cell measurements from blood were acquired from healthy female volunteers in Atlanta, Georgia, participating in a prospective study assessing pharmacokinetics of the antiretroviral drug maraviroc as a candidate HIV prevention drug (ClinicalTrials.gov Identifier: NCT01749566 enrolled 2013–2015). Cellular data used in our analysis were generated from the control arm specimens of the study (not administered drug). Enrollment criteria were that participants were women were aged older than 18 years, HIV-negative, exhibiting normal menses (22–35 days cycle for at least 3 cycles), not using any systemic hormonal contraception or hormonal intrauterine device for at least 6 months prior and were not pregnant or breastfeeding for at least 3 months prior, had no signs or symptoms of vaginal infection at time of screening, had no history of cervical surgeries or procedures, and had no evidence of cardiac or liver disease. The age range of participants were 19–44 years old. Weekly blood specimens were collected at each visit, processed, and analyzed as described below. Progesterone and estradiol were measured from blood plasma using the Milliplex multi-species hormone magnetic bead assay (Millipore Sigma; MSHMAG-21 K) and read on a Bio-Plex® 200 System (Bio-Rad).

### 2.6 Receptor occupancy assay

To measure CCR5 receptor occupancy, PBMC were incubated in the presence or absence of the chemokine MIP-1β (R&D Systems; 271-BME) which binds to CCR5 and inhibits antibody binding and detection of cell surface CCR5 otherwise measured without MIP-1β incubation, as previously described [44]. CCR5 values calculated by using the receptor occupancy assay were determined as the frequency of CCR5-expressing CD4 *T* cells subtracted from those values measured with MIP-1β incubation.

### 2.7 Immune characterizations

Blood was collected in BD Vacutainer® CPT™ cell preparation tubes (CPT) (BD Biosciences; 362,753). Plasma and PBMC were separated by centrifugation using standard procedures and resuspended in saline solution for characterization. Vaginal lavage was performed as previously described [45,46] specimens were collected in 5 ml of sterile phosphate-buffered saline. For extracellular immune characterizations PBMC were stained for viability using Zombie Yellow™ Fixable Viability Kit (Biolegend®; 423,104) then stained using panels of listed fluorochrome conjugated antibodies (Supplemental Tables 1 and 2). For transcription factor measurement, cells were permeabilized using the True Nuclear™ Transcription Factor Buffer Kit (Biolegend®; 424,401) then stained with FoxP3 antibody as indicated by the manufacturer. For intracellular cytokine measurement cells were cultured 5 h in the presence or absence of a cell stimulation cocktail comprising phorbol 12-myristate 13-acetate (PMA), ionomycin, with protein transport inhibitors brefeldin A and monensin (eBioscience™; 00–4970). Following the presence or absence of stimulation, all cells were extracellularly stained then permeabilized using fixation and permeabilization kits prior to intracellular cytokine staining according to the manufacturer instructions (eBioscience™; 88-8824-00). Samples were run on an LSRII flow cytometer. Data was acquired using FACS DIVA software (BD Biosciences) and analyzed using FlowJo software (TreeStar, Inc.).

### 2.8 Cytokine measurement

Soluble cytokines were measured from blood plasma using a bead-based immunoassay kit as indicated by the manufacturer (Biolegend®; legendplex; 740,389). Samples were run on an LSRII flow cytometer. Data was acquired using FACS DIVA software (BD Biosciences) and analyzed using Biolegend® legendplex software.

### 2.9 Periodograms

The Lomb-Scargle Periodogram was calculated for progesterone concentrations and CCR5 expression frequency over time using previously established algorithms [47,48]. The periodogram is calculated by fitting the data with the sinusoidal expression

Asin(2πft)+Bcos(2πft)+C,

where *f* is frequency, *T* is time, and *A, B*, and *C* are fitting parameters determined by minimizing the sum of squared residuals. The fit is performed at many closely spaced frequencies over the range of interest. The Lomb-Scargle power (plotted on the vertical axis) is computed at each frequency as

$$\frac{\chi_{ref}^{2}-\chi^{2}}{\chi_{ref}^{2}}$$

where $\chi_{ref}^{2}$ is the sum of squared residuals around a constant, and $\chi^{2}$ is the sum of squared residuals around the sinusoidal fit. Periodogram peaks indicate sine wave frequencies (inverse periods) appearing most strongly in the data.

### 2.10 Statistical analysis

Cellular immune markers and levels of soluble inflammatory cytokines concentrations by cycle phase were fit using generalized estimating equations (GEE) with an exchangeable working correlation structure to compare repeated measures with continuous variables as previously described [49]. The Chi-square contingency table analysis was used to compare the frequency of concentrations observed in the upper quartile range for IL-1β, IL-6, MCP1, IL-8, TNFα, IFNγ, and the overall mean of type-1 inflammatory cytokines by menstrual cycle phase. The one way ANOVA test was used to detect correlations in repeated measures as previously described [50] using graphpad prism software.

### 2.11 Role of funding source

This study was supported by the U.S. Centers for Disease Control and Prevention, Atlanta, GA 30329 and NIH grants to Emory University (K23AI114407to A.N.S., the Emory University Center for AIDS research [P30AI050409], and Atlanta Clinical and Translational Sciences Institute [KLR2TR000455, UL1TR000454]). The Funders of this study had no role in study design, data collection, data analysis, interpretation, or writing of the manuscript.

## 3 Results

### 3.1 Longitudinal variations in the phenotype and function of CD4 and CD8 *T* cells stratified by phase of the menstrual cycle

**Fig. 1:**
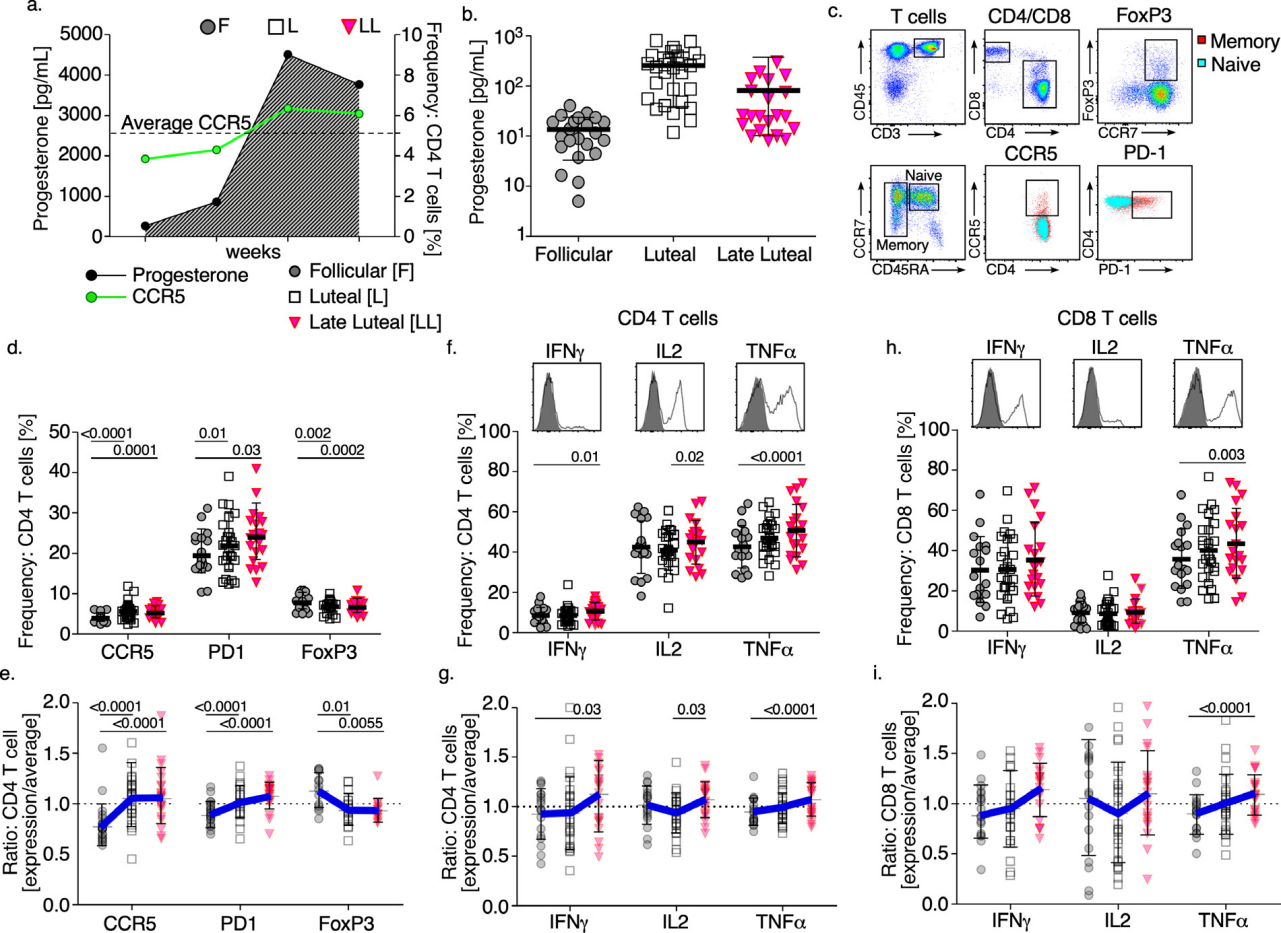
*Longitudinal variations in the phenotype and function of CD4 and CD8 T cells stratified by phase of the menstrual cycle.* (a) Graph from a representative animal depicting weekly progesterone levels (left *y*-axis) and the frequency of CCR5 expression from CD4 *T* cells (right *y*-axis) over the course of one menstrual cycle. The relative phase of the menstrual cycle (labeled top) was estimated by progesterone kinetics. The dotted line designate the average frequency of CCR5 expression on CD4 *T* cells over the course of the cycle. (b) The concentration of P4 stratified by phase of the cycle (c) The gating strategy for CD4 and CD8 *T* cell characterization from PBMC. Viable singlet cells are discriminated for CD45 and CD3 (T cell receptor) expression, lymphocyte size and granularity (not shown), followed by CD4 and CD8 expression in order to measure FoxP3 according to CCR7 signal intensity. To measure CCR5 and PD-1, CD4 *T* cells are further gated for CCR7 and CD45RA to distinguish naïve (further labeled by blue dot plot) versus memory *T* cells (overlaid red dot plot). (d) The frequency of CCR5, PD-1, and FoxP3 expression from CD4 *T* cells measured over the course of one complete menstrual cycle. (e) The fold change of CCR5, PD-1 and FoxP3 expression frequency from CD4 *T* cells at the follicular (gray circle), luteal (open squares), and late luteal (pink triangle) phases measured over one complete menstrual cycle. (f) The frequency of IFNγ, IL-2, and TNFα intracellular cytokine expression from CD4 *T* cells at the indicated phase of the menstrual cycle. (g) The fold change in IFNγ, IL-2, and TNFα intracellular cytokine expression frequency from CD4 *T* cells at the indicated phase of a menstrual cycle. (h) The frequency of IFNγ, IL-2, and TNFα intracellular cytokine expression frequency from CD8 *T* cells at the indicated phase of the menstrual cycle. (i) The fold change in IFNγ, IL-2, and TNFα intracellular cytokine expression frequency from CD8 *T* cells at the indicated phase of the menstrual cycle. (c–i) *Models used to evaluate pairwise comparisons of means were fit using generalized estimating equations* (GEE) (*n* = 9). The p-values considered below the estimated false discovery rate (FDR) for 12 parameters or exhibiting consistency among frequency and fold trends (p-values ≤0.05) are shown (For interpretation of the references to color in this figure legend, the reader is referred to the web version of this article.).

To evaluate immune changes over the menstrual cycle and how this might impact HIV risk, we first compared *T* cell properties from the peripheral blood mononuclear cells (PBMC) of uninfected cycling pig-tailed macaques with progesterone (P4) concentrations in blood plasma. From CD4 *T* cells we monitored CCR5, and PD-1 frequency which are molecules associated with immune activated states [51,52] as well as FoxP3, which is a transcription factor identifying a subtype of CD4 *T* cells (regulatory *T* cells: T_reg_) exhibiting immunosuppressive function [53]. We stratified *T* cell measurements into follicular (lowest P4 levels immediately preceding increases), luteal (rising or peak P4 levels), and late luteal (declining P4 levels post peak) phases of the menstrual cycle (Fig. 1a, b). CD4 *T* cell phenotypes (Fig 1c) stratified by cycle phase presented trends typified by greater activation-associated markers with lower FoxP3 at both the luteal and late luteal phases as compared with the follicular phase (Fig. 1a, d and Supplemental Table 3). The range and average frequency values were as follows: Follicular CCR5 (range: 2.44–6.11, mean: 3.86), PD1 (range: 10.4–31.1, mean: 19.55), and FoxP3 (range: 6.09–10.8, mean: 8.34), Luteal CCR5 (range: 2.43–11.8, mean: 5.59), PD1 (range: 12.4–39, mean: 21.8), and FoxP3 (range: 3.75–10, mean: 6.82), Late Luteal CCR5 (range: 2.78–8.02, mean: 5.22), PD1 (range: 12.8–39, mean: 40.94), and FoxP3 (range: 4.12–10.8, mean: 6.82). Next, we evaluated the expression kinetics over the cycle, by normalizing frequency values taken at any sampling point to the average value measured over that complete cycle (Fig. 1a and Supplemental Fig. 1). By calculating the expression displacement of immune measurements over the course of a menstrual cycle significant trends were observed (Fig. 1e and Supplemental Table 4). CCR5 and PD-1 were more likely to be increased at both the luteal and late luteal phases compared with the follicular phase, while FoxP3 expression was reciprocally decreased.

To analyze *T* cell effector responses to stimulation, we measured intracellular production of key *T* cell cytokines, IFNγ, IL-2, and TNFα from CD4 and CD8 *T* cells (Fig. 1f–i). Comparing mean expression frequencies (Fig. 1f, h and Supplemental Table 3) we found greater CD4 *T* cell IFNγ production from CD4 *T* cells and greater TNFα production from both CD4 and CD8 *T* cells at the late luteal compared with the follicular phase. By also comparing expression kinetics of cytokine production through fold values (as with Fig. 1d) we found that the CD4 *T* cell population exhibited increasing IFNγ and CD4 and CD8 *T* cells exhibited increasing TNFα production during the late luteal phase as compared with the follicular phase (Fig. 1g, i and Supplemental Table 4). These data show that *T* cell population variations in both phenotype and functional responses occur throughout the menstrual cycle which illustrate that elevated P4 throughout the luteal phase accompanies some increased activation-associated properties from CD4 *T* cell populations, though notably, decreasing P4 specifically during the late luteal phase associates with both increased activation-associated properties and increased proinflammatory response to immune stimulation.

### 3.2 Immune variations detected over the course of a menstrual cycle

**Fig. 2:**
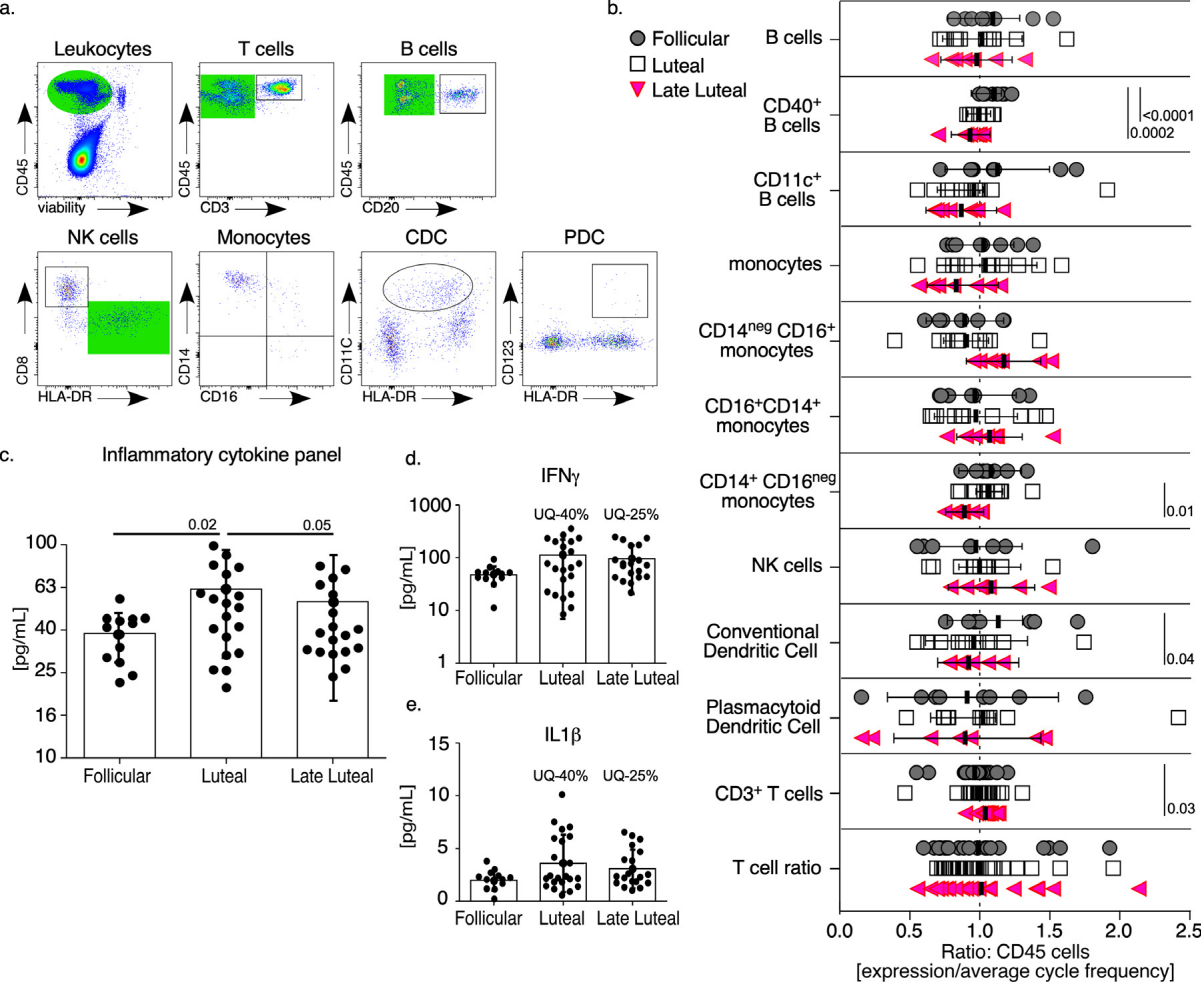
*Immune variations detected over the course of a menstrual cycle.* (a) The gating strategy for immune characterization of PBMC populations. From top left to right, viable leukocytes (green shaded) are distinguished to measure and exclude *T* cells (CD3) and remaining cells (green shaded) are distinguished to measure and exclude *B* cells (CD20). Following *T* and *B* cell exclusion, cells are distinguished for NK cells (HLA-DR.... negative CD8^+^) while HLA-DR....^+^ cells (green shaded) are measured for monocytes frequency (further determined by size and granularity characteristics, not shown). Conventional dendritic cells (CDC) and plasmacytoid dendritic cells (PDC) are measured from CD3 and CD20-negative leukocytes using CD11c^+^ HLA-DR....^+^ gating (CDC) and CD123^+^ HLA-DR....^+^ gating (PDC). (b) PBMC population frequency and indicated phenotypes are measured by phase of the menstrual cycle when collected. (c) Combined mean concentration of inflammatory cytokines detected from blood plasma and stratified by phase of the menstrual cycle when sample was collected. (b, c) Models used to evaluate pairwise comparisons of means were fit using generalized estimating equations (GEE). (d) IFNγ and (e) IL-1β concentrations detected from blood plasma and stratified by phase of the menstrual cycle. (d, e) Frequencies of concentrations contained within the overall upper quartile (UQ) were compared using a Chi-squared test (*n* = 9). (b–e) p-values ≤0.05 are shown (For interpretation of the references to color in this figure legend, the reader is referred to the web version of this article.).

Because *T* cells displayed proinflammatory properties more robustly during the late-luteal phases, we hypothesized that broader innate signaling was regulating this effect [54]. To identify immune signals that could prompt the *T* cell variations observed, we performed a more comprehensive immune characterization of PBMCs, measuring *B* cells, monocytes, NK cells, and conventional or plasmacytoid dendritic cells (DC) as well as the levels of soluble inflammatory cytokines. From PBMC populations (Fig. 2a, b) we found increased *T* cell frequencies at the late luteal phase. Although we did not detect any single population shift that would solely account for this increase, conventional dendritic cells (cDC) were significantly decreased. Although this cDC population are a small proportion of circulating PBMCs and most likely immature, numerical changes in this population have been observed in response to tissue recruitment [55] and these cells have been previously implicated in FRT tissue remodeling during the late luteal phase [56,57].

Immune phenotyping of properties expressed by innate cell populations showed consistent trends with *B* cell and monocyte populations (Fig. 2b). From *B* cells, the mean expression of the humoral or type-2 associated activation molecule, CD40 [58,59] was reduced as the cycle progressed into the luteal and late luteal phase. Monocyte populations typified by either a classical (CD14^+^CD16^neg^), inflammatory (CD14^neg^CD16^+^) or intermediate (CD14^+^CD16^+^) phenotype [60], showed a decrease at the late luteal phase from the predominate classic phenotype as compared with the luteal phase. Although we did not find a corresponding increase in the inflammatory or intermediate populations that was statistically significant, both populations exhibited a mean increase at the late luteal phase, thus accounting for the decreased proportion of classical monocyte populations. Taken together these data show that as the menstrual cycle progresses into the late luteal phase the immune landscape shifts to express increased type-1 cellular properties with corresponding type-2 decreases and this is consistent with a type-1 specific immune response [61].

Due to the type-1 bias we detected from the cellular immune populations we further measured soluble proinflammatory properties over the menstrual cycle, by measuring key cytokines involved in type-1 inflammatory responses (IL-1β, IL-6, IL-8, MCP-1, TNFα, and IFNγ) (Fig. 2c, d, Table 1, and Supplemental Table 5). Using plasma samples collected at follicular, luteal, and late luteal phases, we first stratified measurements as an overall mean value of inflammatory cytokines detected per animal which was increased at the luteal phase (Fig. 2c). Individual cytokine values showed increases in IL-6 at both the luteal and late luteal phase (Supplemental Table 5), however considering the low concentrations of IL-6 detected (Table 1), it is more likely that the increased overall values were not contributed by any distinct cytokine tested. Focusing on the upper quartile of overall cytokine concentrations to compare elevations by phase we found that the upper quartile (UQ) of IFNγ and IL-1β concentrations were detected entirely during the luteal and late luteal phase (Fig. 2d, e and Table 1). During the follicular phase, few specimens contained inflammatory cytokine concentrations within the UQ of the total distribution, however at the luteal and late luteal phase up to 40% of samples were contained within the UQ. Furthermore, for 35% and 25% of samples at the luteal and late luteal phase, respectively, 3 or more inflammatory cytokines had concentrations within their respective UQ (Supplemental Table 6). These data indicate that elevated type-1 proinflammatory signals are detected at the luteal phase and remain elevated as the cycle progresses into late luteal phase.

**Table 1:** Cytokine measurements shown as mean concentration (pg/mL) with the standard deviation (shown in parentheses) at various phases of the menstrual cycle. The frequency of concentrations contained within the overall upper quartile is shown under each cytokine (%). Frequencies were compared using a Chi-square test.

	**Follicular (*n*** **=** **12)**	**Luteal (*n*** **=** **20)**	**Late Luteal (*n*** **=** **20)**	***p*-value (Chi-square)**
**IL-1β**	1.96 (±0.995)	3.83(±2.8)	3.06 (±1.84)	
***upper quartile***	0%	40%	25%	.0210
**IL-6**	1.39 (±0.54)	2.36 (±1.68)	2.01 (±1.06)	
***upper quartile***	0%	35%	30%	.0610
**MCP1**	98.32 (±29.06)	104.07 (±35.02)	100.33 (±27.43)	
***upper quartile***	25%	30%	20%	.8376
**IL-8**	51.99 (±30.23)	63.1 (±47.26)	70.36 (±107.3)	
***upper quartile***	25%	30%	20%	.8376
**TNFα**	2.69 (±4.51)	63.42 (±108.26)	37.76 (±78.79)	
***upper quartile***	0%	35%	30%	.0610
**IFNγ**	47.41 (±19.91)	120.9 (±106.4)	89.56 (±74.56)	
***upper quartile***	0%	40%	25%	.0210
**Overall**	203.79 (±55.9)	357.7 (±208.78)	302.1 (±214.96)	
***upper quartile***	0%	35%	30%	.0610

### 3.3 *T* cell properties at the FRT barrier under menstrual cycle regulation

**Fig. 3:**
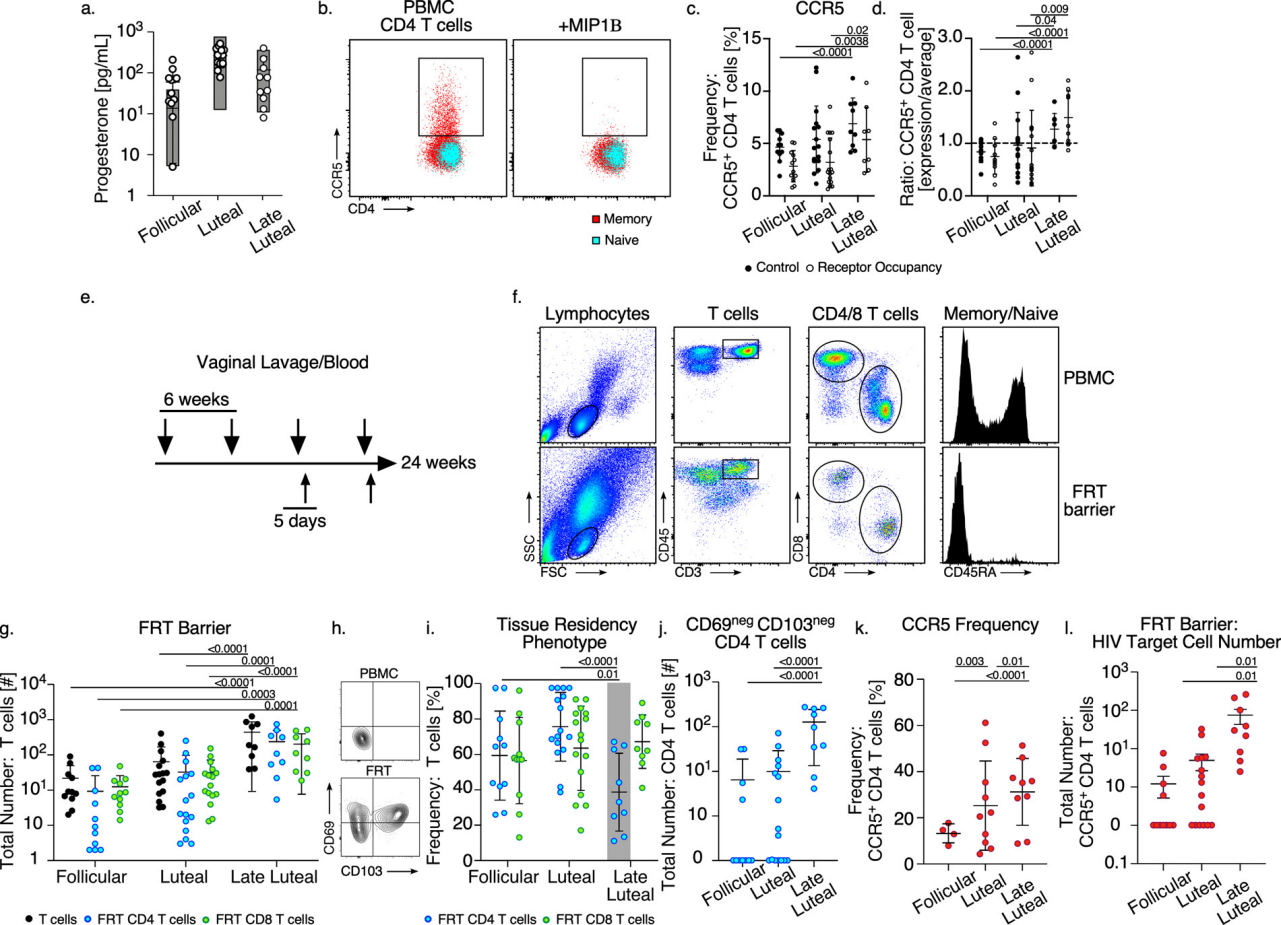
HIV target cell properties at the FRT barrier over the menstrual cycle. (a) The concentration of plasma P4 stratified by phase of the cycle (circles), previous values from Fig 2 are shown as gray floating bars. (b) The cellular gating strategy for measurement of CCR5 from CD4 *T* cells treated in the presence (right panel) or absence of MIP1Β (left panel) from a representative PBMC sample. (c) The CD4 *T* cell surface expression frequency of CCR5 (black dots) or CCR5 values normalized by receptor occupancy (open dots) and stratified by phase of the menstrual cycle at sample collection. (d) The fold change in CD4 *T* cell CCR5 expression frequency (black dots) or CCR5 values normalized by receptor occupancy (open dots) and stratified by phase of the menstrual cycle at sample collection. (e) Schematic depicting the time-course of paired blood and vaginal lavage collection from six typical cycling pig-tailed macaques. (f) The gating strategy for CD4 and CD8 *T* cell characterization from the FRT barrier with paired PBMC from a representative animal. Viable lymphocytes are distinguished by size and granularity and then discriminated for CD45 and CD3 (T cell receptor) expression followed by CD4 and CD8 expression. CD4 *T* cells are further evaluated for CD45RA expression which is not typically expressed on memory *T* cells. (g) The total number of *T* cells (black dots), CD4 *T* cells (blue dots), and CD8 *T* cells (green dots) measured from vaginal lavage sampling and stratified by phase of the menstrual cycle at sample collection. (h) Representative contour plot depicting cell surface CD69 and CD103 expression from PBMC (top panel) or FRT (bottom panel) CD4 *T* cells. (i) The total cell surface expression frequency of CD69 and CD103 from CD4 *T* cells (blue dots) and CD8 *T* cells (green dots) localized at the FRT barrier and stratified by phase of the menstrual cycle at sample collection. (j) The total number of CD4 *T* cells that are negative for CD69 or CD103 expression stratified by phase of the menstrual cycle at sample collection. (k) The cell surface expression frequency of CCR5 from CD4 *T* cells localized at the FRT barrier and stratified by phase of the menstrual cycle at sample collection. (l) The total number of CCR5 expressing CD4 *T* cells detected from lavage sampling of the FRT barrier and stratified by phase of the menstrual cycle at sample collection. (c–l) *Models used to evaluate pairwise comparisons of means were fit using generalized estimating equations* (GEE) (*n* = 6). p-values ≤0.05 are shown (For interpretation of the references to color in this figure legend, the reader is referred to the web version of this article.).

CCR5 signaling facilitates the recruitment of *T* cells into tissue sites experiencing type-1 inflammation including the FRT [62,63]. We hypothesized that the increases in CCR5 expression from circulating CD4 *T* cells corresponded with migratory changes in local *T* cell properties at the site of HIV exposure in the FRT. To test this, we performed FRT barrier sampling in parallel to blood collection from pig-tailed macaques over a period of 24 weeks (Fig. 3). Six animals were measured for weekly plasma progesterone concentrations to confirm typical cycling kinetics (Fig. 3a). PBMC CD4 *T* cells were tested for CCR5 expression with the addition of the receptor occupancy assay for cycle phase comparisons (Fig. 3b, c) [44]. We found that similar to our previous results (Fig. 1), the frequency of CCR5 expression from CD4 *T* cells was greater at the late luteal phase compared with the follicular phase (Fig. 3c and Supplemental Table 7) and this trend was consistent if CCR5 measurements were also normalized by the receptor occupancy assay. Furthermore, by comparing the change in CCR5 expression over time (Fig. 3d and Supplemental Table 8) we found that CCR5 increased at the late luteal phase and this was detected with or without receptor occupancy normalization.

**Fig. 4:**
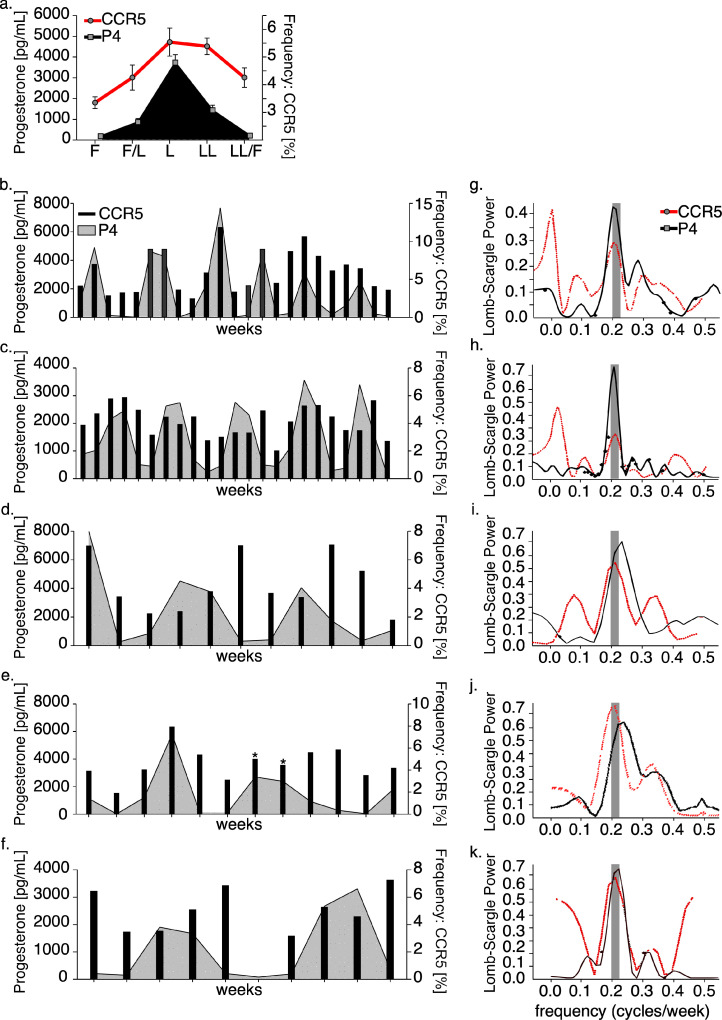
*CCR5 and progesterone oscillations over time.* (a) The mean concentration of detectable progesterone (black histogram) and paired frequency of CCR5 expression on memory CD4 *T* cells (red line) measured from typical cycling macaques (*n* = 9) and stratified by the estimated phase of the cycle when samples were collected. (b–f) Progesterone (gray striped histograms) measurement and paired CCR5 expression on memory CD4 *T* cells (black bars) were measured over time in cycling animals and plotted to depict the longitudinal expression kinetics (*n* = 5). (g–k) The Lomb-Scargle periodograms of progesterone and CCR5 expression from each animal (b–f) with detected oscillations plotted by the Lomb-Scargle power of spectral density. (j) Periodogram calculated to include estimated internal average points (noted by asterisks in e). The peaks in the periodogram indicate the frequencies (inverse periods) of the sine waves appearing most strongly in the data. The gray bars depict the range of periods estimated for the reproductive cycle length of pig-tailed macaques (determined by number of cycles/week) (For interpretation of the references to color in this figure legend, the reader is referred to the web version of this article.).

To investigate FRT barrier *T* cell properties over the menstrual cycle we collected vaginal samples by lavage in parallel to blood samples (Fig. 3e). To prevent tissue perturbation from repeated sampling, collections were taken at 6-week windows such that animals were able to complete a full cycle with staggered sampling time points to include different cycle phases for comparison. After two collection series, a 5-day sampling time point following a 6-week collection was included for a linear cycle phase comparison (Supplemental Fig. 2). *T* cells localized at the FRT epithelial barrier were characterized for expression of cell surface molecules by using paired-PBMC to set cellular gating parameters (Fig. 3f). Quantitation of FRT barrier *T* cells showed that at the late luteal phase, both CD4 and CD8 *T* cell numbers were increased as compared with the follicular and luteal phase (Fig. 3g, Supplemental Table 9 and Supplemental Fig. 2). Because potential changes in mucous viscosity over the menstrual cycle may have influenced lavage collections [64], we also examined FRT *T* cell phenotypic properties that are consistent with tissue residence at immune-restricted sites including the FRT. Previous studies defining the establishment of resident memory *T* cells (TRM) within the epithelial layer of cutaneous tissue identified that CD69, a molecule which functions to inhibit cellular re-entry into circulation through antagonizing lymphatic and blood trafficking signals [65], and CD103 (α-chain of α_E_Β7 integrin epithelial cell adhesion molecule) was unlikely to be expressed on *T* cells that had recently immigrated into the epidermal layer [66,67]. We measured for CD69 and CD103 expression on FRT epithelial barrier *T* cells and compared values by cycle phase (Fig. 3h & i, Supplemental Table 10, and Supplemental Fig. 2). We found that while the FRT *T* cells expressed a high mean frequency of tissue residency phenotypes at the follicular and luteal phase, which is consistent with TRM established within an apical lumen [68], FRT CD4 *T* cell populations expressed reduced mean CD69 levels specifically at the late luteal phase. Furthermore, upon comparing the total number of FRT CD4 *T* cells based upon CD69 and CD103 expression (Fig. 4j and Supplemental Table 11) we found that CD4 *T* cells consistent with recent immigrants (CD69_neg_ CD103_neg_) were most abundant at the late luteal phase compared with the follicular or luteal phase which predominantly yielded CD4 *T* cells expressing TRM phenotypes. Next, we examined HIV target cell populations by measuring CCR5 on FRT CD4 *T* cells (Fig. 3k, l and Supplemental Tables 12, 13) and found that both the frequency and number of HIV target CD4 *T* cells were increased specifically at the late luteal phase. These data show that at the late luteal phase, increased CCR5 expression from CD4 *T* cells in circulation are coupled with increased infiltration of CCR5 expressing CD4 *T* cells into the FRT barrier.

### 3.4 CCR5 and progesterone oscillation lengths over time

**Fig. 5:**
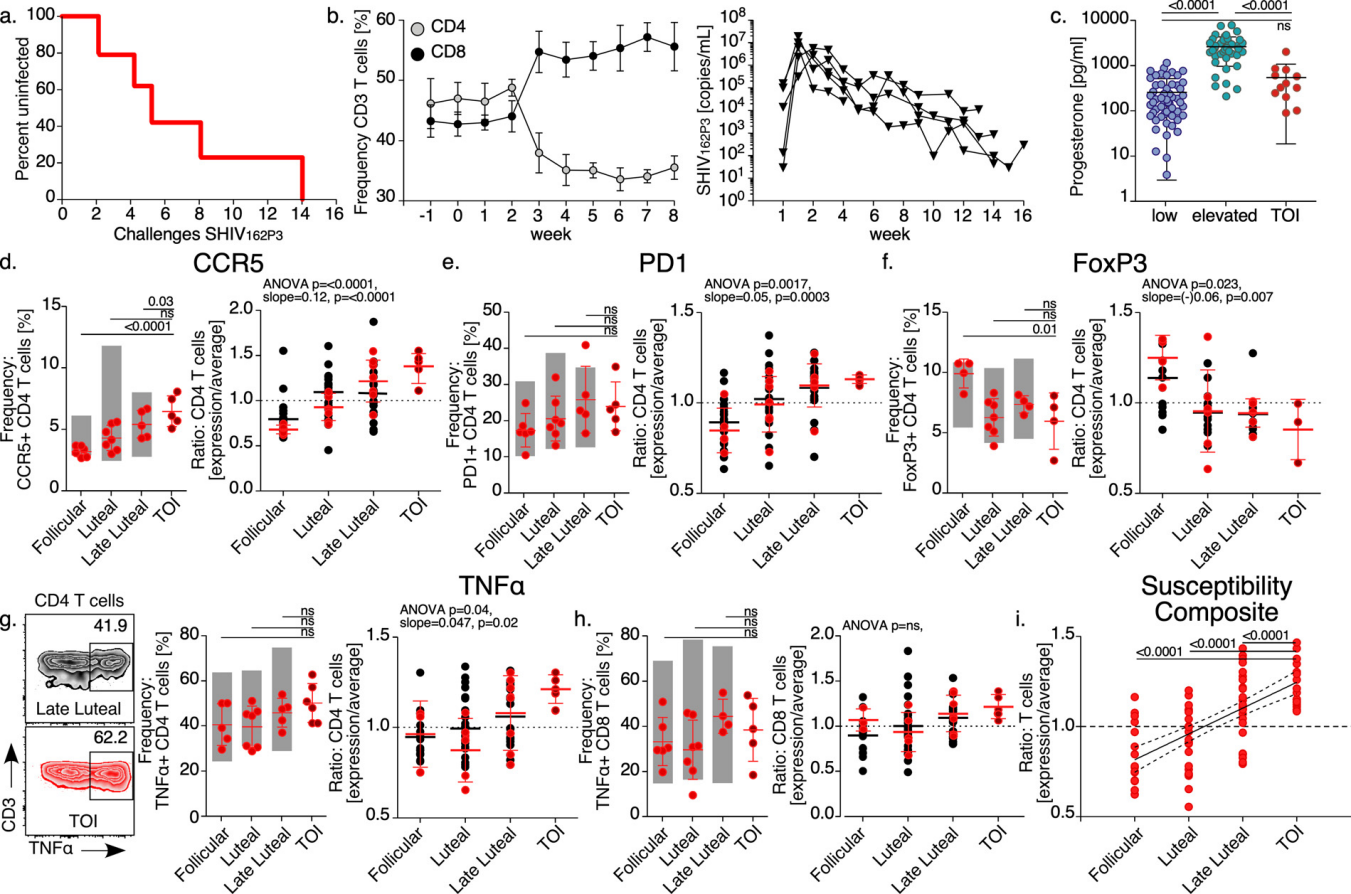
*Immune measurements throughout the menstrual cycle and at the time of infection (TOI) following weekly vaginal SHIV_162P3_ challenge. (*a) Survival curve depicting% of uninfected female macaques (*n* = 6) undergoing challenges with SHIV. (b) (Left panel) frequency of CD4 and CD8 *T* cells in circulation before SHIV infection (week −1), at the time of infection (TOI) (week 0) and following initial infection (week 1–8). (Right panel) plasma SHIV copies/mL (*n* = 5) (c) Progesterone (P4) concentrations in animals grouped by low P4, elevated P4, or TOI of pigtail macaques undergoing SHIV_162P3_ challenge (includes historic data) (*n* = 12). (d–h) Immune marker measurement from uninfected macaques (gray bars or black circles) or macaques undergoing SHIV challenge at the indicated phase of the cycle (red circles shown in d-h, *n* = 5). d (Left panel) CD4 *T* cell CCR5 frequency compared by cycle phase or the TOI. (Right panel) CCR5 expression at indicated phase of the cycle or TOI presented as fold change. (e,f) (Left panel) CD4 *T* cell PD-1 and FoxP3 frequency measured similarly as in (d). (g) (Left panel) contour plots depicting intracellular CD4 *T* cell TNFα production at the late luteal phase or TOI from a single animal undergoing SHIV challenge. Top plot (gray) are TNFα frequency from a late luteal phase challenge not resulting in infection, while bottom plot (red) are levels measured at the TOI. (Center and right panel) TNFα frequency measured and analyzed similarly as in (d–f). (h) TNFα production from CD8 *T* cells measured and analyzed similarly as in (g). (i) Composite of CCR5, PD-1, and TNFα fold change from animals undergoing virus challenge stratified by phase of the menstrual cycle and time of infection (TOI). (c, d**–**h left panel, and i) *Models used to evaluate pairwise comparisons of means were fit using generalized estimating equations* (GEE). (d–h) (right panels) Comparisons across all phases were evaluated using one-way ANOVA followed by a linear trend test (if ANOVA test *p*-values ≤0.05) to determine the slope and linear trend *p*-value (indicated top axis). (i) Linear regression analysis was performed to determine the slope (solid line) and 95% confidence interval (dashed lines above and below the slope). (c–i) *p*-values ≤0.05 are shown (For interpretation of the references to color in this figure legend, the reader is referred to the web version of this article.).

As progesterone levels are critical for regulating reproductive cycles and menstruation, we expanded upon phase estimations to create a composite of CCR5 mean values throughout the menstrual cycle that took into account likely transitional time points (Fig. 4a). This stratification method showed that CCR5 expression displayed a sinusoidal-like pattern over a period of one complete cycle, suggesting that systemic CCR5 oscillations were occurring and were synchronized with menstrual periods. Furthermore, since the immune variations we detected corresponded with cycle phase, we postulated that the menstrual cycle was regulating cellular HIV-risk properties. To test this hypothesis we measured CCR5 and P4 over time in five animals longitudinally for a period of up to 22 weeks. These longitudinal expression patterns showed fluctuations in CCR5 that commonly occurred over a similar period with P4 variation (Fig. 4b–f). To better define these expression trajectories we utilized the Lomb-Scargle periodogram (Fig. 5g–k), a least-squares fit spectral analysis designed to detect and characterize oscillations from uneven or incomplete data sets [47,48]. We first evaluated periodograms from P4 measurements, which detected peak spectral densities at an average frequency of 4.38 weeks, indicating that reproductive periods occurred at an average of 30–31 days (range 26–34.3 days) and this is consistent with the 32.8-day standard cycle of pigtailed macaques [69]. Notably, paired frequency spectrums of CCR5 expression identified sinusoids occurring at an average period of 4.58 weeks or 32 days (range 28–34.3 days). Specifically, two of five animals exhibited CCR5 oscillations occurring at an equal frequency with P4 peaks (Fig. 4g, h), while two animals displayed oscillations occurring at a range of periods within 10% of P4 peaks (Fig. 4i, j). All animals exhibited CCR5 oscillations at frequencies within the standard range of reproductive periods (Fig. 4g–k indicated gray bars) irrespective of progesterone frequencies. These data evidence that type-1 *T* cell oscillations occur in rhythm with the menstrual cycle and are commonly synchronized to progesterone periods.

### 3.5 The immune response throughout the menstrual cycle and at the time of infection (TOI) following weekly vaginal SHIV_162P3_ challenge

By monitoring immune properties over the course of the menstrual cycle in pig-tailed macaques we found that time points of decreasing progesterone are accompanied by increasing type-1 proinflammatory behaviors with increased CCR5 CD4 *T* cell trafficking into the vaginal barrier. To understand the contribution of a type-1 inflammatory shift to HIV susceptibility, we applied the repeat low-dose vaginal SHIV_162P3_ infection model [39] to a subset of animals (*n* = 5) while also monitoring in parallel, circulating immune properties and P4 kinetics in order to identify immune trends at the time of infection (Fig. 5). Weekly administration of virus resulted in productive infections from all 5/5 animals between 2 and 14 weeks of challenge (Fig. 5a) with 3/5 animals remaining uninfected through one or more challenges administered over the late luteal phase of the menstrual cycle.

The acute infection course was consistent among animals as determined by both CD4 *T* cell frequency and virus replication kinetics (Fig. 5b). Because P4 is elevated during tolerogenic timepoints over the menstrual cycle that were suggested to increase HIV infection risk, we examined P4 over the course of a cycle by first stratifying P4 levels as elevated or low (determined by estimating the average P4 value over one complete cycle per animal), and then compared P4 levels to the time of infection (TOI). For this specific analysis we also included historic SHIV infection data in order to better resolve potential trends with P4 levels at the TOI [70] (Fig. 5c). These data show that the TOI was more aligned with challenges that occurred during time points of low P4 concentrations, while challenges during timepoints associated with heightened immune suppression were unlikely to result in infection.

Next, we asked whether the cellular abundance of HIV risk properties was linked to infection by comparing immune frequencies between the estimated TOI and phases throughout the cycle when infection did not occur (Fig. 5d–f, h far left panel, Fig. 5g center panel). These mean frequency comparisons showed greater CCR5 expression with less FoxP3 expression at the TOI compared with the follicular phase indicating that the frequency of CCR5 and FoxP3 expression at the TOI was not consistent with the CCR5 and FoxP3 levels measured during the follicular phase. CCR5 expression at the TOI was also greater when compared with the late luteal phase though the range of CCR5 frequency values at TOI fell within the expected ranges at the luteal and late luteal phases from both challenged and unchallenged animals (ranges taken from Fig. 1 measurements included as floating bars for comparisons). Additionally, no differences in PD-1 or TNFα production were found when comparing the TOI with different cycle phases. These data indicate that although the frequency of *T* cells expressing susceptibility markers at the TOI are more consistent with levels observed at the luteal and late phase, these measures did not account for the lack of productive SHIV infection seen in some challenges occurring during those phases.

Because the inflammatory immune properties displayed oscillations synchronized to the menstrual cycle phase, we asked whether the change in immune molecule expression from the *T* cell population over the course of an oscillation might provide stronger indications of SHIV infection risk (Fig. 5d–h, right panels of figures). Using linear trend tests to compare *T* cell properties at the TOI with timepoints within the menstrual cycle that did not result in infection (left panel of figures), we found that the TOI consistently occurred during a state of increasing CCR5, PD-1, and TNFα expression from CD4 *T* cells while the immunosuppressive marker, FoxP3 was decreasing. To further understand this pattern we combined *T* cell susceptibility characteristics (CCR5, PD-1, and TNFα) into a composite analysis (Fig. 5i) and found that the TOI singularly corresponded with rising *T* cell inflammatory properties. These data show that the TOI is linked by the slope of *T* cell inflammatory expression kinetics and these mounting immune responses are most likely to occur within the late luteal phase when P4 levels are decreased.

### 3.6 *T* cell immune measurements over the human menstrual cycle

**Fig. 6:**
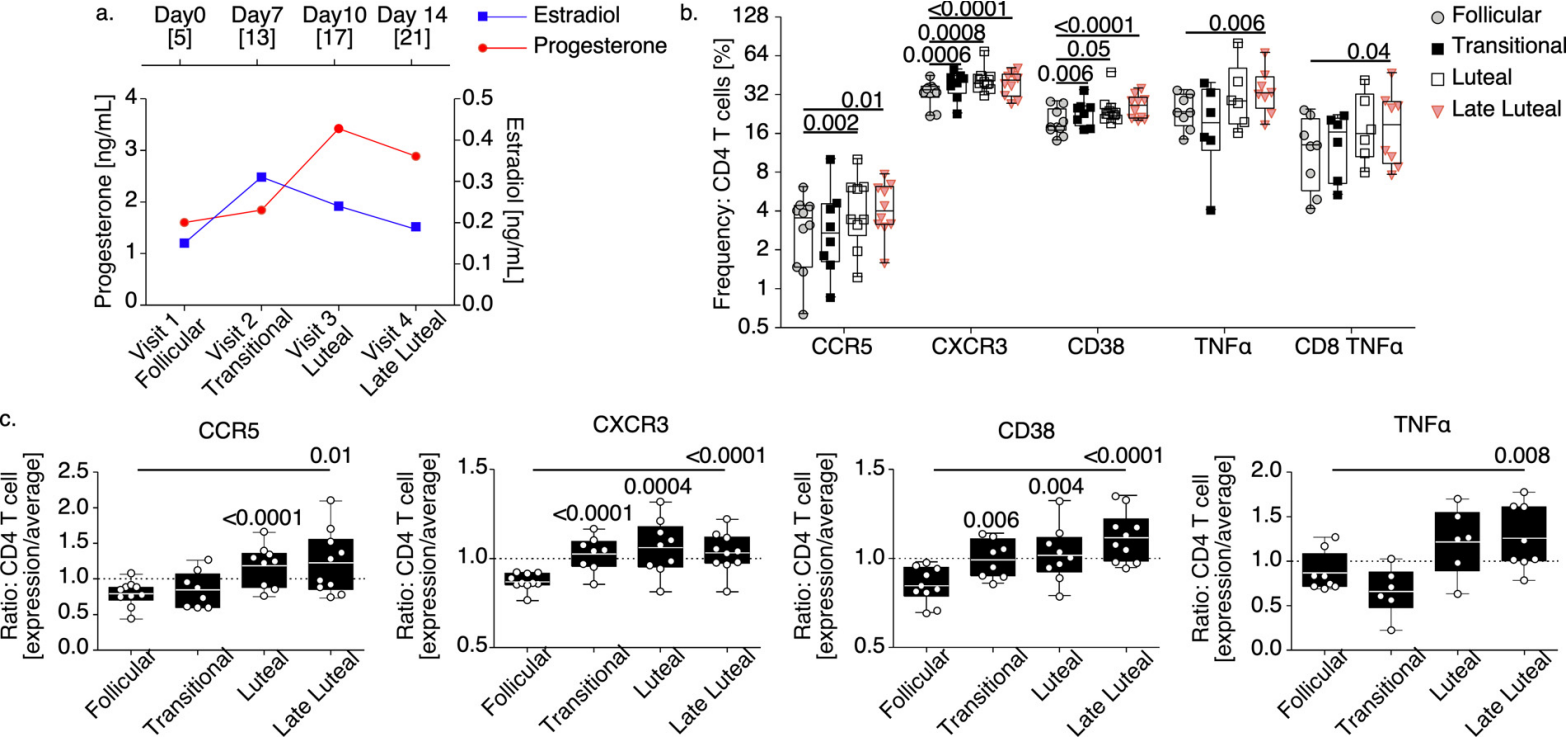
*Coordinated menstrual cycle kinetics and T cell immune measurements in healthy women.***(**a) The mean concentration of detectable progesterone (red lines) and estradiol (blue lines) measured longitudinally from typical cycling women (*n* = 10). Sampling visits were timed according to phase of the menstrual cycle (top axis label indicates spacing of visits with the average actual day of the cycle at visit [shown in brackets]; average day determined by date of menses). (b) The frequency of indicated immune molecule expression on CD4 *T* cells or TNFα intracellular expression from CD8 *T* cells (as labeled) stratified according to the phase of the menstrual cycle. (c) Relative expression of the indicated immune molecules on CD4 *T* cells at specific phases of the menstrual cycle and normalized by the average expression over one complete cycle. (b, c) *Models used to evaluate pairwise comparisons (to the follicular phase) of means were fit using generalized estimating equations* (GEE). The *p*-values considered below the estimated false discovery rate (FDR) for 9 parameters or exhibiting consistency among frequency and fold trends (*p*-values ≤0.05) are shown for comparisons with the follicular phase (For interpretation of the references to color in this figure legend, the reader is referred to the web version of this article.).

To determine whether cycle-based variations in circulating HIV risk factors occur in humans we measured CD4 *T* cell characteristics in 10 healthy, typical-cycling women. These women were participants in a study assessing the pharmacokinetics and immune effects of the HIV treatment drug Maraviroc (Fig. 6), and were included in a control, untreated arm. PBMC and plasma was collected over the course of a menstrual cycle with sampling visits set according to reported status of menses such that follicular phase, relative time of ovulation (labeled transitional), early luteal, and late luteal phases could be more stringently identified (Fig. 6a). We measured CCR5 and intracellular cytokine production from *ex-vivo* immune stimulation similarly as with macaques, however for activation-associated markers we used CD38, which has been previously linked with HIV susceptibility [11,71] as well as CXCR3, a chemokine receptor that similar to CCR5 regulates *T* cell trafficking into peripheral sites of type-1 inflammation[72] (Fig. 6b and Supplemental Table 14). As compared with the follicular phase, we discovered greater mean CCR5 and CD38 CD4 *T* cell expression at the luteal and late luteal phases. CXCR3 increased at all time points following the follicular phase while the frequency of TNFα producing CD4 *T* cells increased from the follicular phase solely at the late luteal phase. No differences comparing IFNγ or IL-2 by cycle phase were found (data not shown).

By comparing the change in HIV-risk properties over the course of a menstrual cycle, we observed mounting expression in all markers at the late luteal phase (Fig. 6c and Supplemental Table 15). Specifically, following the follicular phase an increase in all CD4 risk properties CCR5, CD38, CXCR3, and TNFα was identified uniquely at the late luteal phase. These data demonstrate that similar to our findings in macaques, a type-1 inflammatory response occurs over the menstrual cycle in humans and support that increased HIV susceptibility is most likely to occur during the late luteal phase.

## 4 Discussion

The menstrual cycle is linked with the exacerbation of common autoimmune diseases and increased susceptibility to certain infections including HIV, yet the mechanisms underlying this relationship remain poorly understood [73]. In this study we identified a coordinated immune response that are consistent with a type-1 specific inflammatory bias within the luteal phase of the menstrual cycle. In particular we found fluctuations in CCR5 which play a vital role in regulating *T* cell type-1 inflammatory effector responses as well as being a required co-receptor for HIV [74]. Corresponding with this response we found increased trafficking of CCR5 CD4 *T* cells at the FRT barrier occurring most often at the late luteal phase. These data support that in preparation for menstruation at the late luteal phase, the menstrual cycle induce the recruitment of HIV target cells, which increase infection risk following sexual exposure.

To test whether the type-1 inflammatory shift during the late luteal phase of menstrual cycle influence HIV infection, we used the low-dose repeat SHIV vaginal challenge model in cycling pig-tailed macaques and in parallel monitored systemic HIV-risk properties. By comparing the time of infection (TOI) with challenges that did not result in a productive infection, we found no overall difference in the frequency of immune properties at the late luteal phase or other cycle phases save an increased mean expression of CCR5. However, the frequency range of all *T* cell properties measured including CCR5 at the TOI, fell within the expected range we observed from our immune measurements in unchallenged animals. This suggest that although the abundance of HIV-risk properties are increased at the late luteal phase, this increase did not directly predict infection in SHIV-challenged animals or account for why infections did not occur from challenges within this time frame. Notably, upon comparing the kinetic sequence of immune properties over the virus challenge course with the TOI, we discovered a consistent increasing trajectory at the TOI. These data evidence that mounting type-1 *T* cell inflammatory responses that occur in parallel with the immune functions mediating the process of menstruation within the late luteal phase were critical for infection to occur from vaginal challenge. Taken together these data describe a model for HIV susceptibility under menstrual cycle regulation whereby upon entering the luteal phase of the menstrual cycle, the absence of fertilization leads to progesterone withdrawal and a decrease in immunosuppressive immune properties which initiates the late luteal phase. The production of inflammatory signals from the FRT in preparation for menstruation further leads to an increase in target *T* cell recruitment and opportunities for HIV infection.

As the menstrual cycle is not the result of an infection, it is reasonable to suppose that a lack of archetypal CD4 *T* cell activation through antigen-specific recognition indicates little to no change within the target *T* cell population that might increase HIV infection risk in the FRT. However, accumulating work on “bystander” *T* cell activation demonstrates that type-1 inflammatory signals are sufficient to stimulate certain effector responses from *T* cells in the absence of TCR signaling, in particular non-cognate CD4 *T* cell trafficking into inflamed tissues sites [75,76]. Furthermore, previous work on the programming of tissue resident *T* cells found that recently immigrated *T* cells within some epithelial barrier sites do not express tissue retention properties such as CD69 and maintain the ability to respond to migratory cues that would facilitate reentry from the tissues back into the circulation through afferent lymphatic trafficking [66,67,[77], [78], [79]]. It is therefore possible that recently immigrated target cells at sites of virus exposure are an important route by which HIV can gain access into the circulation and establish a systemic infection.

In regard to other HIV risk factors in women such as progestin-based hormonal contraception [[80], [81], [82]] and STI's, associated immune activation and inflammation are commonalities that likely drive HIV susceptibility. Yet, because progestin-based HCs modulate the progesterone signaling pathway distinctly from the menstrual cycle [80] and the immune responses to STIs are a result of antigenic recognition, it is difficult to make mechanistic comparisons with our findings on the menstrual cycle to these other important susceptibility factors. Moreover previous work evaluating systemic immune markers and HIV acquisition report diverse and complex immune signaling associated with increased risk [83,84]. Yet all of these aforementioned risk factors likely influence susceptibility through HIV target cell modulation, which indicate that an emphasis on the intrinsic regulation of CD4 *T* cell immune properties might better inform upon detectable and potentially uniform markers of increased HIV risk.

To conclude, our study provides valuable new insights into the role of the immune response in vaginal HIV transmission under menstrual cycle regulation. We define for the first time, to our knowledge, periodic shifts in type-1 inflammatory behaviors that are associated with increased susceptibility to HIV infection through target cell recruitment into the site of pathogen exposure.

We posit that novel HIV prevention strategies that reduce local inflammation events in the FRT, such as occurs under menstrual cycle regulation, may help prevent HIV transmission events in women [85].

## Author contributions

ASK performed experiments, analyzed, and interpreted data. FH performed statistical and modeling analysis. JB and EWB designed, analyzed, and interpreted periodogram data. ASK, IM, and JGGL designed the NHP study. AS, ASK, and IO designed the human study. SS performed and analyzed cytokine testing. JB, AS, FH, SS, EWB, IO, and IM provided critical advice and helped with the interpretation and discussion of the results. ASK and JGGL wrote the manuscript, with contributions from all authors.

## Data sharing statement

Access to data generated and analyzed in this study will be provided upon reasonable request to the corresponding author.

## Declaration of Competing Interest

The authors declare no competing interests.

## Appendix Supplementary materials

Image, image 1

Image, image 2

Image, application 3

Image, application 4

Image, application 5

Image, application 6

Image, application 7

Image, application 8

Image, application 9

Image, application 10

Image, application 11

Image, application 12

Image, application 13

Image, application 14

Image, application 15

Image, application 16

Image, application 17

Image, application 18

Image, application 19
