# Supplementary material for: Proinflammatory oscillations over the menstrual cycle drives bystander CD4 T cell recruitment and SHIV susceptibility from vaginal challenge
Source: eBioMedicine. 2021 Jul 3;69:103472. doi: 10.1016/j.ebiom.2021.103472 (PMC8264117; doi:10.1016/j.ebiom.2021.103472)
Supplement: Supplementary file 1 [file mmc1.docx]

**Supplementary Material**

**
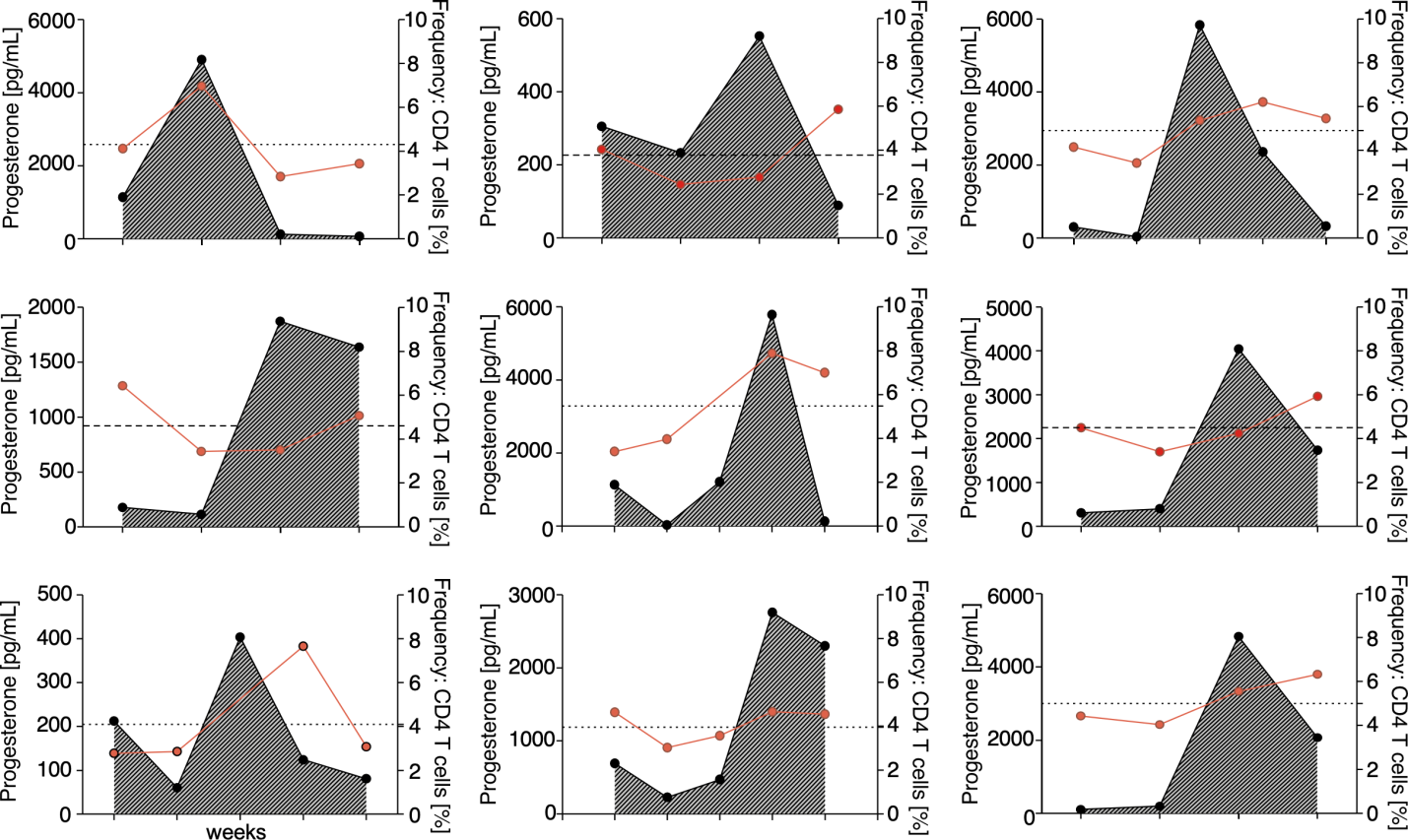
**

**Supplementary Figure 1: CD4 T cell CCR5 expression frequency over the course of a menstrual cycle in pig-tailed macaques.** A representative graph from each animal depicting weekly progesterone measurements (gray shaded histogram, left y-axis) and the frequency of CCR5 expression from CD4 T cells (red line, right y-axis).

**
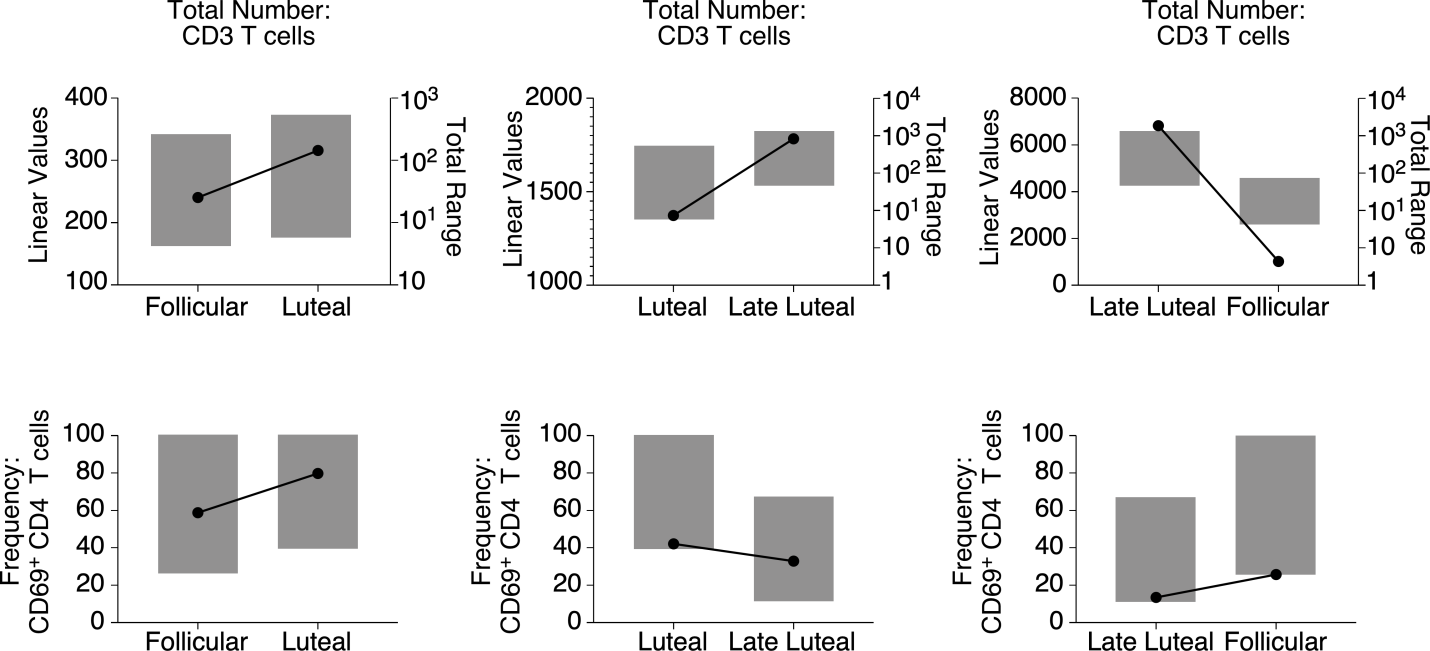
**

**Supplementary Figure 2: Linear immune measurements.** The total number of FRT T cells, (Top 3 panels) and CD69 expression from FRT CD4 T cells (bottom 3 panels) are depicted by sample collection 5 days apart (black lines) and stratified by the animals’ phase of the menstrual cycle at sample collection. The range of values measured at indicated phase of the menstrual cycle from 6-week sample collections are shown as underlaid floating grey bars (right y-axis for top panels). Linear measurements, follicular to luteal (N=6), Luteal to late luteal (N=1), and late luteal to follicular (N=1) are displayed as a before-after graph plot.
