# Supplementary material for: Proinflammatory oscillations over the menstrual cycle drives bystander CD4 T cell recruitment and SHIV susceptibility from vaginal challenge
Source: eBioMedicine. 2021 Jul 3;69:103472. doi: 10.1016/j.ebiom.2021.103472 (PMC8264117; doi:10.1016/j.ebiom.2021.103472)
Supplement: Supplementary file 2 [file mmc2.docx]

|  | antibody | conjugate | clone | catalogue | vendor |
| --- | --- | --- | --- | --- | --- |
| 1. | CD3 | Alexa Fluor® 700 | SP34-2 | 557917 | BD Biosciences |
| 2. | CD4 | Brilliant Violet 785™ | OKT4 | 317442 | Biolegend® |
| 3. | CD8 | V500 | RPA-T8 | 560774 | BD Biosciences |
| 4. | CD11c | Brilliant Violet 711™ | 3.9 | 301630 | Biolegend® |
| 5. | CD14 | APC | M5E2 | 301808 | Biolegend® |
| 6. | CD16 | PE-CF594 | 3G8 | 562293 | BD Biosciences |
| 7. | CD20 | FITC | 2H7 | 555622 | BD Biosciences |
| 8. | CD40 | PE-Cy™7 | 5C3 | 334322 | Biolegend® |
| 9. | CD45 | BV421 | D058-1283 | 740084 | BD Biosciences |
| 10. | CD45RA | PE-Cy™7 | 5H9 | 561216 | BD Biosciences |
| 11. | CD69 | APC/Fire™ 750 | FN50 | 310946 | Biolegend® |
| 12. | CD103 | FITC | Ber-ACT8 | 550259 | BD Biosciences |
| 13. | CD123 | PE | 6H6 | 306006 | Biolegend® |
| 14. | CCR5 | BV650 | 3A9 | 564999 | BD Biosciences |
| 15. | CCR7 | PE-CF594 | 150503 | 562381 | BD Biosciences |
| 16. | PD1 | Brilliant Violet 711™ | EH12.2H7 | 329928 | Biolegend® |
| 17. | HLADR | BV786 | G46-6 | 564041 | BD Biosciences |
| 18. | FoxP3 | PE | 206D | 320108 | Biolegend® |
| 19. | IFNγ | Brilliant Violet 650™ | 45.B3 | 563416 | BD Biosciences |
| 20. | IL-2 | Brilliant Violet 605™ | MQ1-17H12 | 500332 | Biolegend® |
| 21. | TNFα | APC | Mab11 | 502912 | Biolegend® |
