# Supplementary material for: Proinflammatory oscillations over the menstrual cycle drives bystander CD4 T cell recruitment and SHIV susceptibility from vaginal challenge
Source: eBioMedicine. 2021 Jul 3;69:103472. doi: 10.1016/j.ebiom.2021.103472 (PMC8264117; doi:10.1016/j.ebiom.2021.103472)
Supplement: Supplementary file 3 [file mmc3.docx]

|  | antibody | conjugate | clone | catalogue | vendor |
| --- | --- | --- | --- | --- | --- |
| 1. | CD3 | APC-H7 | SK7 | 560176 | BD Biosciences |
| 2. | CD4 | Alexa Fluor® 700 | RPA-T4 | 557922 | BD Biosciences |
| 3. | CD8a | Brilliant Violet 510™ | RPA-T8 | 301048 | Biolegend® |
| 4. | CD45RA | Brilliant Violet 605™ | HI100 | 304134 | Biolegend® |
| 5. | CXCR3 | PerCp/Cyanine5.5 | G025H7 | 353714 | Biolegend® |
| 6. | CCR5 | PE | 3A9 | 556042 | BD Biosciences |
| 7. | CCR7 | PE-CF594 | 150503 | 562381 | BD Biosciences |
| 8. | CD38 | PE-Cy™7 | HB7 | 335790 | BD Biosciences |
| 9. | IFNγ | APC | B27 | 554702 | BD Biosciences |
| 10. | IL-2 | PE-Cy™7 | MQ1-17H12 | 560707 | BD Biosciences |
| 11. | TNFα | FITC | Mab11 | 554512 | BD Biosciences |
