# Supplementary material for: Proinflammatory oscillations over the menstrual cycle drives bystander CD4 T cell recruitment and SHIV susceptibility from vaginal challenge
Source: eBioMedicine. 2021 Jul 3;69:103472. doi: 10.1016/j.ebiom.2021.103472 (PMC8264117; doi:10.1016/j.ebiom.2021.103472)
Supplement: Supplementary file 4 [file mmc4.docx]

| Figure | predictor  (Frequency value) | Comparison | Mean Difference | Lower 95% | | Upper 95% | p value |
| --- | --- | --- | --- | --- | --- | --- | --- |
| Fig 1d | CCR5+ CD4 T cells | Follicular with Late Luteal | -1.3632 | | -2.0336 | -0.6928 | 0.0001 |
|  |  | Follicular with Luteal | -1.736 | | -2.4859 | -0.9861 | <0.0001 |
|  |  | Luteal with Late Luteal | 0.3728 | | -0.8569 | 1.6026 | 0.5524 |
|  | PD-1+ CD4 T cells | Follicular with Late Luteal | -4.1791 | | -8.033 | -0.3252 | 0.0336 |
|  |  | Follicular with Luteal | -2.2578 | | -4.0973 | -0.4183 | 0.0161 |
|  |  | Luteal with Late Luteal | -1.9213 | | -6.1099 | 2.2672 | 0.3686 |
|  | FoxP3+ CD4 T cells | Follicular with Late Luteal | 1.5148 | | 0.7247 | 2.3048 | 0.0002 |
|  |  | Follicular with Luteal | 1.5139 | | 0.5395 | 2.4883 | 0.0023 |
|  |  | Luteal with Late Luteal | 0.0009 | | -0.7301 | 0.7319 | 0.9981 |
|  |  |  |  | |  |  |  |
| Fig 1e | IFNγ+ CD4 T cells | Follicular with Late Luteal | -1.9185 | | -3.4473 | -.3898 | 0.0139 |
|  |  | Follicular with Luteal | 0.0349 | | -3.5074 | 3.5772 | 0.9846 |
|  |  | Luteal with Late Luteal | -1.9534 | | -4.5499 | 0.646 | 0.1403 |
|  | IL2+ CD4 T cells | Follicular with Late Luteal | -2.6577 | | -11.121 | 5.8059 | 0.5383 |
|  |  | Follicular with Luteal | 1.3394 | | -8.903 | 11.608 | 0.7982 |
|  |  | Luteal with Late Luteal | -3.9971 | | -7.3517 | -0.6424 | 0.0195 |
|  | TNFα+ CD4 T cells | Follicular with Late Luteal | -8.4048 | | -11.67 | -5.1392 | <0.0001 |
|  |  | Follicular with Luteal | -4.6577 | | -11.335 | 2.0197 | 0.1716 |
|  |  | Luteal with Late Luteal | -3.7471 | | -10.085 | 2.5909 | 0.2466 |
|  |  |  |  | |  |  |  |
| Fig 1f | IFNγ+ CD8 T cells | Follicular with Late Luteal | -4.1511 | | -11.336 | 3.0337 | 0.2575 |
|  |  | Follicular with Luteal | -0.0562 | | -7.6404 | 7.5279 | 0.9884 |
|  |  | Luteal with Late Luteal | -4.0949 | | -10.013 | 1.8233 | 0.1751 |
|  | IL2+ CD8 T cells | Follicular with Late Luteal | -0.5506 | | -2.5644 | 1.4632 | 0.592 |
|  |  | Follicular with Luteal | 0.2318 | | -2.3821 | 2.8456 | 0.862 |
|  |  | Luteal with Late Luteal | -0.7824 | | -3.438 | 1.8733 | 0.5637 |
|  | TNFα+ CD8 T cells | Follicular with Late Luteal | -6.9426 | | -11.522 | -2.3634 | 0.003 |
|  |  | Follicular with Luteal | -4.1043 | | -11.055 | 2.846 | 0.2471 |
|  |  | Luteal with Late Luteal | -2.8383 | | -9.2935 | 3.6169 | 0.3888 |
|  |  |  |  | |  |  |  |
|  |  |  |  | |  |  |  |
|  |  |  |  | |  |  |  |
|  |  |  |  | |  |  |  |
