# Supplementary material for: Proinflammatory oscillations over the menstrual cycle drives bystander CD4 T cell recruitment and SHIV susceptibility from vaginal challenge
Source: eBioMedicine. 2021 Jul 3;69:103472. doi: 10.1016/j.ebiom.2021.103472 (PMC8264117; doi:10.1016/j.ebiom.2021.103472)
Supplement: Supplementary file 5 [file mmc5.docx]

| **Figure** | **predictor (fold value)** | **Comparison** | **Mean difference** | **Lower 95%** | **Upper 95%** | **p value** |
| --- | --- | --- | --- | --- | --- | --- |
| Fig 1g | CCR5+ CD4 T cells | Follicular with Late Luteal | -3.3463 | -0.4845 | -0.2081 | <0.0001 |
|  |  | Follicular with Luteal | -0.3362 | -0.4549 | -0.2174 | <0.0001 |
|  |  | Luteal with Late Luteal | -0.0101 | -0.2343 | 0.2141 | 0.9296 |
|  | PD-1+ CD4 T cells | Follicular with Late Luteal | -0.2014 | -0.3004 | -0.1023 | 0.0001 |
|  |  | Follicular with Luteal | -0.1395 | -0.1823 | -0.0967 | <0.0001 |
|  |  | Luteal with Late Luteal | -0.0619 | -0.1364 | -0.0127 | 0.104 |
|  | FoxP3+ CD4 T cells | Follicular with Late Luteal | 0.2062 | 0.0605 | 0.3519 | 0.0055 |
|  |  | Follicular with Luteal | 0.2012 | 0.0478 | 0.3546 | 0.0102 |
|  |  | Luteal with Late Luteal | 0.005 | -0.1015 | 0.1116 | 0.9263 |
|  |  |  |  |  |  |  |
| Fig 1h | IFNγ+ CD4 T cells | Follicular with Late Luteal | -0.2152 | -0.4126 | -0.0158 | 0.0344 |
|  |  | Follicular with Luteal | -0.015 | -0.3702 | 0.3401 | 0.9338 |
|  |  | Luteal with Late Luteal | -0.1991 | -0.5035 | 0.1052 | 0.1997 |
|  | IL2+ CD4 T cells | Follicular with Late Luteal | -0.0629 | -0.2397 | 0.1139 | 0.4858 |
|  |  | Follicular with Luteal | 0.0742 | -0.1572 | 0.3057 | 0.5296 |
|  |  | Luteal with Late Luteal | -0.1371 | -0.2601 | -0.0141 | 0.0289 |
|  | TNFα+ CD4 T cells | Follicular with Late Luteal | -0.1449 | -0.219 | -0.0708 | 0.0001 |
|  |  | Follicular with Luteal | -0.0676 | -0.1993 | 0.064 | 0.3139 |
|  |  | Luteal with Late Luteal | -0.0772 | -0.2259 | 0.0715 | 0.3088 |
|  |  |  |  |  |  |  |
| Fig 1i | IFNγ+ CD8 T cells | Follicular with Late Luteal | -0.1974 | -0.3577 | -0.0371 | 0.0158 |
|  |  | Follicular with Luteal | -0.0225 | -0.2415 | 0.1964 | 0.8401 |
|  |  | Luteal with Late Luteal | -0.1749 | -0.3216 | -0.0282 | 0.0195 |
|  | IL2+ CD8 T cells | Follicular with Late Luteal | -0.1195 | -0.3319 | 0.0920 | 0.2701 |
|  |  | Follicular with Luteal | 0.1068 | -0.2251 | 0.4387 | 0.5282 |
|  |  | Luteal with Late Luteal | -0.2263 | -0.4068 | -0.0459 | 0.0139 |
|  | TNFα+ CD8 T cells | Follicular with Late Luteal | -0.1982 | -0.2658 | -0.1306 | <0.0001 |
|  |  | Follicular with Luteal | -0.1088 | -0.2851 | 0.0676 | 0.2268 |
|  |  | Luteal with Late Luteal | -0.0895 | -0.2403 | 0.0613 | 0.2449 |
