# Supplementary material for: Proinflammatory oscillations over the menstrual cycle drives bystander CD4 T cell recruitment and SHIV susceptibility from vaginal challenge
Source: eBioMedicine. 2021 Jul 3;69:103472. doi: 10.1016/j.ebiom.2021.103472 (PMC8264117; doi:10.1016/j.ebiom.2021.103472)
Supplement: Supplementary file 6 [file mmc6.docx]

| **Cytokine** | **Comparison** | **Difference** | **LCL** | **UCL** | **p-value** |
| --- | --- | --- | --- | --- | --- |
| IL1β | Follicular with Luteal | -0.426 | -1.052 | 0.2 | 0.182 |
|  | Follicular with Late Luteal | -0.154 | -0.58 | .271 | 0.477 |
|  | Luteal with Late Luteal | -0.271 | -0.719 | 0.177 | 0.235 |
| IL6 | Follicular with Luteal | -0.636 | -1.269 | -0.003 | 0.049 |
|  | Follicular with Late Luteal | -0.41 | -0.803 | -0.016 | 0.041 |
|  | Luteal with Late Luteal | -0.226 | -0.72 | 0.267 | 0.369 |
| MCP1 | Follicular with Luteal | -0.555 | -1.148 | 0.037 | 0.066 |
|  | Follicular with Late Luteal | -0.501 | -0.993 | -0.01 | 0.046 |
|  | Luteal with Late Luteal | -0.054 | -0.425 | 0.317 | 0.775 |
| IL8 | Follicular with Luteal | -0.167 | -0.887 | 0.554 | 0.65 |
|  | Follicular with Late Luteal | 0.126 | -0.403 | 0.656 | 0.641 |
|  | Luteal with Late Luteal | -0.293 | -0.917 | 0.331 | 0.358 |
| TNFα | Follicular with Luteal | -0.406 | -0.958 | 0.146 | 0.15 |
|  | Follicular with Late Luteal | -0.235 | -0.494 | 0.024 | 0.075 |
|  | Luteal with Late Luteal | -0.171 | -0.791 | 0.45 | 0.59 |
| IFNγ | Follicular with Luteal | -0.565 | -1.204 | 0.075 | 0.084 |
|  | Follicular with Late Luteal | -0.262 | -0.562 | 0.037 | 0.086 |
|  | Luteal with Late Luteal | -0.302 | -0.821 | 0.216 | 0.253 |
| **Overall** | Follicular with Luteal | -0.677 | -1.234 | -0.12 | 0.017 |
|  | Follicular with Late Luteal | -0.181 | -0.639 | 0.277 | 0.439 |
|  | Luteal with Late Luteal | -0.496 | -0.982 | -0.011 | 0.045 |
|  |  |  |  |  |  |
|  |  |  |  |  |  |
|  |  |  |  |  |  |
|  |  |  |  |  |  |
|  |  |  |  |  |  |
|  |  |  |  |  |  |
|  |  |  |  |  |  |
