# Supplementary material for: Proinflammatory oscillations over the menstrual cycle drives bystander CD4 T cell recruitment and SHIV susceptibility from vaginal challenge
Source: eBioMedicine. 2021 Jul 3;69:103472. doi: 10.1016/j.ebiom.2021.103472 (PMC8264117; doi:10.1016/j.ebiom.2021.103472)
Supplement: Supplementary file 7 [file mmc7.docx]

| **cytokines** | **Follicular (n=12)** | **Luteal (n=20)** | **Late Luteal (n=20)** |
| --- | --- | --- | --- |
| 0 | 58% | 30% | 50% |
| 1 | 33% | 20% | 15% |
| 2 | 8.30% | 15% | 5% |
| 3 | 0% | 0% | 5% |
| 4 | 0% | 10% | 10% |
| 5 | 0% | 20% | 15% |
| 6 | 0% | 5% | 0% |
|  |  |  |  |
| ≤3 | 100% | 65% | 75% |
| >3 | 0% | 35% | 25% |
