# Supplementary material for: Proinflammatory oscillations over the menstrual cycle drives bystander CD4 T cell recruitment and SHIV susceptibility from vaginal challenge
Source: eBioMedicine. 2021 Jul 3;69:103472. doi: 10.1016/j.ebiom.2021.103472 (PMC8264117; doi:10.1016/j.ebiom.2021.103472)
Supplement: Supplementary file 8 [file mmc8.docx]

| **Figure** | **predictor (frequency value)** | **Comparison** | **Mean difference** | **Lower 95%** | **Upper 95%** | **p value** |
| --- | --- | --- | --- | --- | --- | --- |
| Fig 3c | CCR5^+^CD4 T cells | Follicular with Luteal | -0.7434 | -1.7325 | 0.2456 | 0.1407 |
|  |  | Follicular with Late Luteal | -2.2497 | -3.3311 | -1.1683 | <0.0001 |
|  |  | Luteal with Late Luteal | -1.5063 | -3.2009 | 0.1884 | 0.0815 |
|  | receptor occupancy | Follicular with Luteal | -0.4839 | -1.4333 | 0.4654 | 0.3178 |
|  |  | Follicular with Late Luteal | -2.43 | -4.0778 | -0.7822 | 0.0038 |
|  |  | Luteal with Late Luteal | -1.9461 | -3.6165 | -0.2756 | 0.0224 |
|  |  |  |  |  |  |  |
|  |  |  |  |  |  |  |
|  |  |  |  |  |  |  |
|  |  |  |  |  |  |  |
|  |  |  |  |  |  |  |
|  |  |  |  |  |  |  |
|  |  |  |  |  |  |  |
|  |  |  |  |  |  |  |
|  |  |  |  |  |  |  |
|  |  |  |  |  |  |  |
|  |  |  |  |  |  |  |
|  |  |  |  |  |  |  |
|  |  |  |  |  |  |  |
|  |  |  |  |  |  |  |
|  |  |  |  |  |  |  |
|  |  |  |  |  |  |  |
|  |  |  |  |  |  |  |
|  |  |  |  |  |  |  |
|  |  |  |  |  |  |  |
|  |  |  |  |  |  |  |
|  |  |  |  |  |  |  |
|  |  |  |  |  |  |  |
