# Supplementary material for: Proinflammatory oscillations over the menstrual cycle drives bystander CD4 T cell recruitment and SHIV susceptibility from vaginal challenge
Source: eBioMedicine. 2021 Jul 3;69:103472. doi: 10.1016/j.ebiom.2021.103472 (PMC8264117; doi:10.1016/j.ebiom.2021.103472)
Supplement: Supplementary file 9 [file mmc9.docx]

| **Figure** | **predictor (fold value)** | **Comparison** | **Mean difference** | **Lower 95%** | **Upper 95%** | **p value** |
| --- | --- | --- | --- | --- | --- | --- |
| Fig 3d | CCR5^+^CD4 T cells | Follicular with Luteal | -0.1278 | -0.3455 | 0.09 | 0.2502 |
|  |  | Follicular with Late Luteal | -0.4279 | -0.6075 | -0.2482 | <0.0001 |
|  |  | Luteal with Late Luteal | -0.3001 | -0.5804 | -0.0198 | 0.0359 |
|  | receptor occupancy | Follicular with Luteal | -0.1605 | -0.4769 | 0.156 | 0.3202 |
|  |  | Follicular with Late Luteal | -0.7359 | -1.0971 | -0.3747 | <0.0001 |
|  |  | Luteal with Late Luteal | -0.5754 | -1.0087 | -0.1421 | 0.0092 |
|  |  |  |  |  |  |  |
|  |  |  |  |  |  |  |
|  |  |  |  |  |  |  |
|  |  |  |  |  |  |  |
|  |  |  |  |  |  |  |
|  |  |  |  |  |  |  |
|  |  |  |  |  |  |  |
|  |  |  |  |  |  |  |
|  |  |  |  |  |  |  |
|  |  |  |  |  |  |  |
|  |  |  |  |  |  |  |
|  |  |  |  |  |  |  |
|  |  |  |  |  |  |  |
|  |  |  |  |  |  |  |
|  |  |  |  |  |  |  |
|  |  |  |  |  |  |  |
|  |  |  |  |  |  |  |
|  |  |  |  |  |  |  |
|  |  |  |  |  |  |  |
|  |  |  |  |  |  |  |
|  |  |  |  |  |  |  |
|  |  |  |  |  |  |  |
