# Supplementary material for: Proinflammatory oscillations over the menstrual cycle drives bystander CD4 T cell recruitment and SHIV susceptibility from vaginal challenge
Source: eBioMedicine. 2021 Jul 3;69:103472. doi: 10.1016/j.ebiom.2021.103472 (PMC8264117; doi:10.1016/j.ebiom.2021.103472)
Supplement: Supplementary file 10 [file mmc10.docx]

| **Figure** | **predictor (total number)** | **Comparison** | **Mean difference** | **Lower 95%** | **Upper 95%** | **p value** |
| --- | --- | --- | --- | --- | --- | --- |
| Fig 3g | T cells | Follicular with Luteal | -443.511 | -1394.1 | 507.09 | 0.3605 |
|  |  | Follicular with Late Luteal | -5487.08 | -7736.4 | -3237.8 | <0.0001 |
|  |  | Luteal with Late Luteal | -5043.57 | -6758.5 | -3328.7 | <0.0001 |
|  | CD4 T cells | Follicular with Luteal | -232.359 | -629.52 | 164.805 | 0.2515 |
|  |  | Follicular with Late Luteal | -2296.83 | -3530.3 | -1063.4 | <0.0001 |
|  |  | Luteal with Late Luteal | -2064.48 | -3076.2 | -1052.7 | 0.0001 |
|  | CD8 T cells | Follicular with Luteal | -190.044 | -473.20 | 93.1135 | 0.1884 |
|  |  | Follicular with Late Luteal | -1901.77 | -2856.7 | -946.88 | 0.0001 |
|  |  | Luteal with Late Luteal | -1711.73 | -2442.6 | -980.83 | <0.0001 |
|  |  |  |  |  |  |  |
|  |  |  |  |  |  |  |
|  |  |  |  |  |  |  |
|  |  |  |  |  |  |  |
|  |  |  |  |  |  |  |
|  |  |  |  |  |  |  |
|  |  |  |  |  |  |  |
|  |  |  |  |  |  |  |
|  |  |  |  |  |  |  |
|  |  |  |  |  |  |  |
|  |  |  |  |  |  |  |
|  |  |  |  |  |  |  |
|  |  |  |  |  |  |  |
|  |  |  |  |  |  |  |
|  |  |  |  |  |  |  |
|  |  |  |  |  |  |  |
|  |  |  |  |  |  |  |
|  |  |  |  |  |  |  |
|  |  |  |  |  |  |  |
