# Supplementary material for: Proinflammatory oscillations over the menstrual cycle drives bystander CD4 T cell recruitment and SHIV susceptibility from vaginal challenge
Source: eBioMedicine. 2021 Jul 3;69:103472. doi: 10.1016/j.ebiom.2021.103472 (PMC8264117; doi:10.1016/j.ebiom.2021.103472)
Supplement: Supplementary file 11 [file mmc11.docx]

| **Figure** | **predictor (frequency value)** | **Comparison** | **Mean difference** | **Lower 95%** | **Upper 95%** | **p value** |
| --- | --- | --- | --- | --- | --- | --- |
| Fig 3i | CD69^+^ CD103^+^CD4 T cells | Follicular with Luteal | -16.6562 | -37.118 | 3.8053 | 0.1106 |
|  |  | Follicular with Late Luteal | 22.1304 | 4.045 | 40.2158 | 0.0017 |
|  |  | Luteal with Late Luteal | 38.7866 | 30.8839 | 46.6893 | <0.0001 |
|  | CD69^+^ CD103^+^ CD8 T cells | Follicular with Luteal | -6.9182 | -15.175 | 1.339 | 0.1006 |
|  |  | Follicular with Late Luteal | -1.496 | -15.257 | 12.2547 | 0.8311 |
|  |  | Luteal with Late Luteal | 5.4222 | -12.375 | 23.2195 | 0.5504 |
|  |  |  |  |  |  |  |
|  |  |  |  |  |  |  |
|  |  |  |  |  |  |  |
|  |  |  |  |  |  |  |
|  |  |  |  |  |  |  |
|  |  |  |  |  |  |  |
|  |  |  |  |  |  |  |
|  |  |  |  |  |  |  |
|  |  |  |  |  |  |  |
|  |  |  |  |  |  |  |
|  |  |  |  |  |  |  |
|  |  |  |  |  |  |  |
|  |  |  |  |  |  |  |
|  |  |  |  |  |  |  |
|  |  |  |  |  |  |  |
|  |  |  |  |  |  |  |
|  |  |  |  |  |  |  |
|  |  |  |  |  |  |  |
|  |  |  |  |  |  |  |
|  |  |  |  |  |  |  |
|  |  |  |  |  |  |  |
|  |  |  |  |  |  |  |
