# Supplementary material for: Proinflammatory oscillations over the menstrual cycle drives bystander CD4 T cell recruitment and SHIV susceptibility from vaginal challenge
Source: eBioMedicine. 2021 Jul 3;69:103472. doi: 10.1016/j.ebiom.2021.103472 (PMC8264117; doi:10.1016/j.ebiom.2021.103472)
Supplement: Supplementary file 13 [file mmc13.docx]

| **Figure** | **predictor (frequency value)** | **Comparison** | **Mean difference** | **Lower 95%** | **Upper 95%** | **p value** |
| --- | --- | --- | --- | --- | --- | --- |
| Fig 3k | CCR5^+^ CD4 T cells | Follicular with Luteal | -10.9923 | -18.246 | -3.7396 | 0.003 |
|  |  | Follicular with Late Luteal | -26.4119 | -33.723 | -19.1 | <0.0001 |
|  |  | Luteal with Late Luteal | -15.4193 | -27.666 | -3.1724 | 0.0136 |
|  |  |  |  |  |  |  |
|  |  |  |  |  |  |  |
|  |  |  |  |  |  |  |
|  |  |  |  |  |  |  |
|  |  |  |  |  |  |  |
|  |  |  |  |  |  |  |
|  |  |  |  |  |  |  |
|  |  |  |  |  |  |  |
|  |  |  |  |  |  |  |
|  |  |  |  |  |  |  |
|  |  |  |  |  |  |  |
|  |  |  |  |  |  |  |
|  |  |  |  |  |  |  |
|  |  |  |  |  |  |  |
|  |  |  |  |  |  |  |
|  |  |  |  |  |  |  |
|  |  |  |  |  |  |  |
|  |  |  |  |  |  |  |
|  |  |  |  |  |  |  |
|  |  |  |  |  |  |  |
|  |  |  |  |  |  |  |
|  |  |  |  |  |  |  |
