# Supplementary material for: Proinflammatory oscillations over the menstrual cycle drives bystander CD4 T cell recruitment and SHIV susceptibility from vaginal challenge
Source: eBioMedicine. 2021 Jul 3;69:103472. doi: 10.1016/j.ebiom.2021.103472 (PMC8264117; doi:10.1016/j.ebiom.2021.103472)
Supplement: Supplementary file 14 [file mmc14.docx]

| **Figure** | **predictor (total number)** | **Comparison** | **Mean difference** | **Lower 95%** | **Upper 95%** | **p value** |
| --- | --- | --- | --- | --- | --- | --- |
| Fig 3l | CCR5^+^ CD4 T cells | Follicular with Luteal | -36.3926 | -93.277 | 20.4919 | 0.2099 |
|  |  | Follicular with Late Luteal | -732.197 | -1301.9 | -162.52 | 0.0118 |
|  |  | Luteal with Late Luteal | -695.805 | -1225.0 | -166.61 | 0.01 |
|  |  |  |  |  |  |  |
|  |  |  |  |  |  |  |
|  |  |  |  |  |  |  |
|  |  |  |  |  |  |  |
|  |  |  |  |  |  |  |
|  |  |  |  |  |  |  |
|  |  |  |  |  |  |  |
|  |  |  |  |  |  |  |
|  |  |  |  |  |  |  |
|  |  |  |  |  |  |  |
|  |  |  |  |  |  |  |
|  |  |  |  |  |  |  |
|  |  |  |  |  |  |  |
|  |  |  |  |  |  |  |
|  |  |  |  |  |  |  |
|  |  |  |  |  |  |  |
|  |  |  |  |  |  |  |
|  |  |  |  |  |  |  |
|  |  |  |  |  |  |  |
|  |  |  |  |  |  |  |
|  |  |  |  |  |  |  |
|  |  |  |  |  |  |  |
