# Supplementary material for: Proinflammatory oscillations over the menstrual cycle drives bystander CD4 T cell recruitment and SHIV susceptibility from vaginal challenge
Source: eBioMedicine. 2021 Jul 3;69:103472. doi: 10.1016/j.ebiom.2021.103472 (PMC8264117; doi:10.1016/j.ebiom.2021.103472)
Supplement: Supplementary file 15 [file mmc15.docx]

| **Figure** | **predictor (frequency value)** | **Comparison** | **Mean difference** | **Lower 95%** | **Upper 95%** | **p value** |
| --- | --- | --- | --- | --- | --- | --- |
| Fig 6b | CCR5+ CD4 T cells | Follicular with Transitional | -0.3115 | -1.5005 | 0.8775 | 0.6076 |
|  |  | Follicular with Luteal | -1.374 | -2.2347 | -0.5133 | 0.0018 |
|  |  | Follicular with Late Luteal | -1.224 | -2.1621 | -0.2859 | 0.0105 |
|  | CD38+ CD4 T cells | Follicular with Transitional | -3.72 | -6.3842 | -1.0558 | 0.0062 |
|  |  | Follicular with Luteal | -2.8644 | -5.72 | -0.0089 | 0.0493 |
|  |  | Follicular with Late Luteal | -6.16 | -8.6412 | -3.6788 | <0.0001 |
|  | CXCR3+ CD4 T cells | Follicular with Transitional | -5.5475 | -8.7198 | -2.3752 | 0.0006 |
|  |  | Follicular with Luteal | -9.1767 | -14.564 | -3.7885 | 0.0008 |
|  |  | Follicular with Late Luteal | -6.04 | -8.4374 | -3.6426 | <0.0001 |
|  | TNFα+ CD4 T cells | Follicular with Transitional | 2.9058 | -5.8955 | 11.7072 | 0.5176 |
|  |  | Follicular with Luteal | -11.0208 | -25.563 | 3.522 | 0.1375 |
|  |  | Follicular with Late Luteal | -11.375 | -19.422 | -3.3282 | 0.0056 |
|  | TNFα+ CD8 T cells | Follicular with Transitional | -1.3954 | -8.3978 | 5.607 | 0.6961 |
|  |  | Follicular with Luteal | -7.0004 | -17.039 | 3.0383 | 0.1717 |
|  |  | Follicular with Late Luteal | -7.5338 | -14.859 | -0.2086 | 0.0438 |
|  |  |  |  |  |  |  |
|  |  |  |  |  |  |  |
|  |  |  |  |  |  |  |
|  |  |  |  |  |  |  |
|  |  |  |  |  |  |  |
|  |  |  |  |  |  |  |
|  |  |  |  |  |  |  |
|  |  |  |  |  |  |  |
|  |  |  |  |  |  |  |
|  |  |  |  |  |  |  |
|  |  |  |  |  |  |  |
|  |  |  |  |  |  |  |
|  |  |  |  |  |  |  |
