# Supplementary material for: Proinflammatory oscillations over the menstrual cycle drives bystander CD4 T cell recruitment and SHIV susceptibility from vaginal challenge
Source: eBioMedicine. 2021 Jul 3;69:103472. doi: 10.1016/j.ebiom.2021.103472 (PMC8264117; doi:10.1016/j.ebiom.2021.103472)
Supplement: Supplementary file 16 [file mmc16.docx]

| **Figure** | **predictor (fold value)** | **Comparison** | **Mean Difference** | **Lower 95%** | **Upper 95%** | **p value** |
| --- | --- | --- | --- | --- | --- | --- |
| Fig 6c | CCR5+ CD4 T cells | Follicular with Transitional | -0.0521 | -0.2289 | 0.1247 | 0.5635 |
|  |  | Follicular with Luteal | -0.3953 | -0.5396 | -0.251 | <0.0001 |
|  |  | Follicular with Late Luteal | -0.4345 | -0.7759 | -0.0931 | 0.0126 |
|  | CD38+ CD4 T cells | Follicular with Transitional | -0.1467 | -0.251 | -0.0425 | 0.0058 |
|  |  | Follicular with Luteal | -0.172 | -0.2876 | -0.0565 | 0.0035 |
|  |  | Follicular with Late Luteal | -0.2708 | -0.3851 | -0.1565 | <0.0001 |
|  | CXCR3+ CD4 T cells | Follicular with Transitional | -0.1564 | -0.2299 | -0.0829 | <0.0001 |
|  |  | Follicular with Luteal | -0.1918 | -0.2984 | -0.0853 | 0.0004 |
|  |  | Follicular with Late Luteal | -0.163 | -0.2266 | -0.0994 | <0.0001 |
|  | TNFα+ CD4 T cells | Follicular with Transitional | 0.2124 | -0.1114 | 0.5362 | 0.1985 |
|  |  | Follicular with Luteal | -0.3488 | -0.64 | -0.0576 | 0.0189 |
|  |  | Follicular with Late Luteal | -0.384 | -0.6682 | -0.0997 | 0.0081 |
|  |  |  |  |  |  |  |
|  |  |  |  |  |  |  |
|  |  |  |  |  |  |  |
|  |  |  |  |  |  |  |
|  |  |  |  |  |  |  |
|  |  |  |  |  |  |  |
|  |  |  |  |  |  |  |
|  |  |  |  |  |  |  |
|  |  |  |  |  |  |  |
|  |  |  |  |  |  |  |
|  |  |  |  |  |  |  |
|  |  |  |  |  |  |  |
|  |  |  |  |  |  |  |
|  |  |  |  |  |  |  |
|  |  |  |  |  |  |  |
|  |  |  |  |  |  |  |
