# Supplementary material for: Proinflammatory oscillations over the menstrual cycle drives bystander CD4 T cell recruitment and SHIV susceptibility from vaginal challenge
Source: eBioMedicine. 2021 Jul 3;69:103472. doi: 10.1016/j.ebiom.2021.103472 (PMC8264117; doi:10.1016/j.ebiom.2021.103472)
Supplement: Supplementary file 17 [file mmc17.docx]

**Research in context:**

**Evidence before this study**

Understanding the contribution of the menstrual cycle to STI/HIV infection from sexual exposure may help prevent new infections, and inform upon other potential risk factors in women such as hormonal contraception. Exposure to HIV in the female reproductive tract (FRT) during fertility windows has been previously proposed to increase infection susceptibility through heightened immune tolerance. Although proinflammatory responses and the proximity of CCR5+ CD4 T cells (HIV target cells) to mucosal exposure sites are considered HIV-risk factors, when and how the immune system is regulated by the menstrual cycle to influence infection in the FRT remains undefined.

**Added value of this study:**

We identify in humans and pig-tailed macaques that the menstrual cycle regulates a type-1 specific adaptive immune response, identified in part by increased CD4 T cell expression of proinflammatory properties and the HIV co-receptor CCR5 within the late luteal phase. Furthermore, in macaques we find that the sinusoidal fluctuations of CCR5+ memory CD4 T cells in circulation corresponds with infiltration of CCR5+ CD4 T cells into the vaginal mucosa primarily within the late luteal phase of the cycle, when SHIV inoculation also led to the establishment of infection from weekly repetitive low-dose vaginal challenge.

**Implications of all the available evidence:**

This study finds evidence that over the menstrual cycle HIV-risk from sexual exposure is increased by the FRT tissue remodeling functions that occur during the late luteal phase of the menstrual cycle.
